# Polymer-Based Biomaterials in Periodontal and Peri-Implant Soft Tissue Augmentation: Biological Rationale, Clinical Applications, and Future Perspectives

**DOI:** 10.3390/polym18182272

**Published:** 2026-09-17

**Authors:** Bartłomiej Górski, Natalia Muczkowska

**Affiliations:** 1 Department of Periodontology and Oral Diseases, Medical University of Warsaw, 02-097 Warsaw, Poland; 2 Department of Oral Surgery, Medical University of Warsaw, 02-097 Warsaw, Poland

**Keywords:** polymer biomaterials, collagen matrix, hyaluronic acid, hydrogels, periodontal plastic surgery, peri-implant soft tissues, connective tissue graft, tissue engineering, regenerative dentistry, biomaterials

## Abstract

Soft tissue deficiencies around teeth and dental implants remain a significant challenge in contemporary periodontology and implant dentistry. These deficiencies influence a number of factors, including esthetic outcomes, peri-implant tissue stability, long-term maintenance, and patient satisfaction. Although autogenous connective tissue grafts remain the clinical gold standard for soft tissue augmentation, their use is limited by donor-site morbidity, increased surgical time, and restricted tissue availability. Consequently, polymer-based biomaterials have emerged as promising alternatives or adjunctive regenerative materials capable of improving wound healing while reducing surgical invasiveness. This narrative review compares natural polymers, including collagen, hyaluronic acid, extracellular matrix-derived scaffolds, fibrin-based platelet concentrates, and gelatin-based materials, with synthetic polymeric systems, such as polycaprolactone, polylactic acid, polyethylene glycol, and advanced composite hydrogels. The influence of these materials on angiogenesis, fibroblast migration, extracellular matrix remodeling, immune modulation, and soft tissue integration is reviewed in the context of periodontal plastic surgery and peri-implant soft tissue reconstruction. This narrative review summarizes the current landscape of polymer-based biomaterials used for periodontal and peri-implant soft tissue augmentation, integrating material science with clinical evidence. The advantages, limitations, indications and future perspectives of polymer-based substitutes are discussed in comparison with autogenous grafting procedures.

## 1. Introduction

Soft tissue augmentation is essential for contemporary periodontal and implant therapy. Beyond aesthetics, the quality and quantity of soft tissue play a key role in maintaining periodontal health, facilitating plaque control, enhancing comfort, and ensuring long-term stability of restorations and implants. Inadequate soft tissue, thin tissue, insufficient keratinized tissue, gingival recession and peri-implant tissue deficiencies increase susceptibility to mucosal recession, impaired hygiene, peri-implant inflammation and compromised esthetics. Contemporary treatment concepts emphasize not only hard tissue reconstruction but also the optimization of soft tissue around implants and periodontal health [1,2,3,4].

Autogenous soft tissue grafting, particularly with connective tissue grafts (CTGs) and free gingival grafts (FGGs), is the clinical gold standard for periodontal and peri-implant soft tissue augmentation. However, harvesting autogenous tissue necessitates a secondary surgical site and is associated with increased operative time, postoperative pain, bleeding, limited tissue availability, and donor-site morbidity. Alternative biomaterials with the capacity to decrease surgical invasiveness while achieving outcomes comparable to those of conventional methods are being extensively researched [5,6,7,8].

Recent research shows that polymer-based materials are particularly promising for regenerating soft tissue around implants. Unlike conventional graft substitutes, modern biopolymers increasingly serve as biologically active scaffolds, modulating tissue regeneration through interactions with the immune system and resident cells [9,10,11,12].

**Figure 1:**
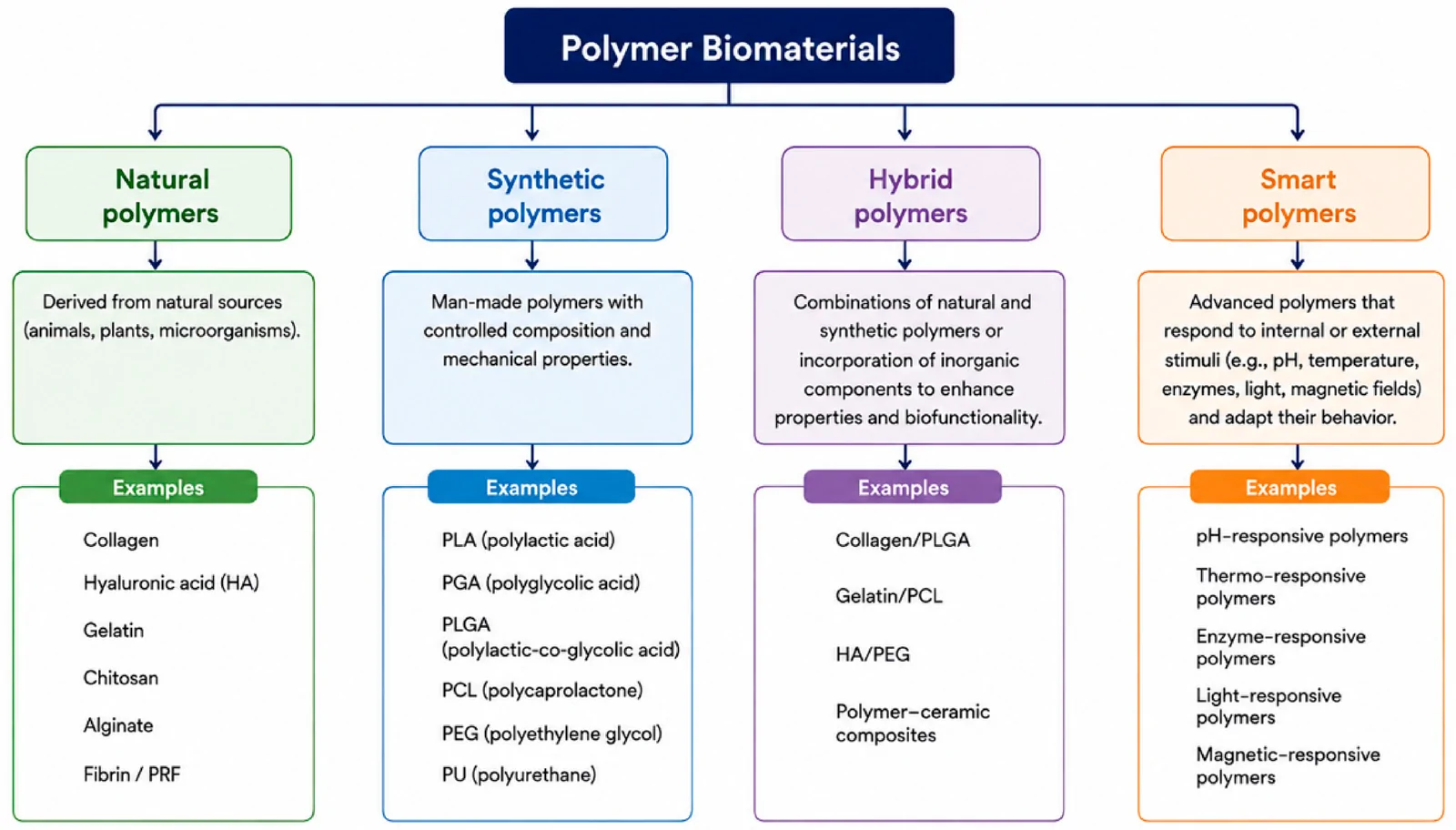
Classification of polymer-based biomaterials used in periodontal and peri-implant soft tissue regeneration. Four categories are shown, each with its defining description and representative examples. Natural polymers (green) are derived from natural sources, including animals, plants and microorganisms; examples include collagen, hyaluronic acid, gelatin, chitosan, alginate, and fibrin or platelet-rich fibrin. Synthetic polymers (blue) are manufactured with controlled composition and mechanical properties; examples include polylactic acid (PLA), polyglycolic acid (PGA), poly(lactic-co-glycolic acid) (PLGA), polycaprolactone (PCL), polyethylene glycol (PEG), and polyurethane (PU). Hybrid polymers (purple) combine natural and synthetic components, or incorporate inorganic phases, to enhance properties and biofunctionality; examples include collagen/PLGA, gelatin/PCL, HA/PEG, and polymer–ceramic composites. Smart polymers (orange) respond to internal or external stimuli such as pH, temperature, enzymatic activity, and light or magnetic fields and adapt their behavior accordingly. Color-coding is used consistently for the four categories throughout the figure.

Polymeric biomaterials utilized in soft tissue regeneration encompass both natural and synthetic materials. Natural polymers are biocompatible and have been demonstrated to enhance angiogenesis, fibroblast proliferation, and collagen synthesis, thereby promoting wound healing. In contrast, synthetic polymers provide greater control over mechanical properties, degradation rates, structural architecture, and manufacturing reproducibility [13,14,15] (Figure 1).

Hybrid biomaterials combine the biological advantages of natural matrices with improved mechanical performance and structural stability. Contemporary polymeric constructs now function as regenerative platforms capable of controlling cellular behavior, modulating inflammation, promoting neovascularization, and serving as carriers for factors, agents, extracellular vesicles, or stem cells. The emergence of injectable hydrogels, electrospun nanofibers, biofunctionalized collagen matrices, 3D-printed scaffolds, and smart stimuli-responsive polymers illustrates the rapid evolution of this field [16,17,18,19,20,21].

Despite rapid technological advances, translation of polymer-based biomaterials into routine clinical practice remains challenging. Many commercially available materials have shown encouraging biological properties in preclinical studies, but their clinical benefits are modest compared to autogenous connective tissue grafts. Substantial heterogeneity also exists in polymer composition, crosslinking methods, degradation kinetics, porosity, mechanical behavior, and biological performance, so that clinicians often face uncertainty regarding the optimal selection of biomaterials for specific clinical indications.

The aim of this narrative review is to provide a comprehensive overview of polymer-based biomaterials used for soft tissue augmentation. Particular emphasis is placed on the relationships between polymer structure, physicochemical properties, host biological response, and clinical performance. Emerging technologies are discussed to provide clinicians and researchers with an evidence-based framework for future regenerative therapy.

Literature was identified through PubMed, Scopus, Web of Science and the Cochrane Library for English-language publications from 2000 to 2026, combining terms for soft tissue augmentation, connective tissue graft, collagen matrix, acellular dermal matrix, hyaluronic acid, chitosan, alginate, fibrin and platelet concentrates, synthetic polymers including polylactic acid, polyglycolic acid, poly(lactic-co-glycolic acid), polycaprolactone, polyethylene glycol and polyurethane, hybrid and composite scaffolds, keratinized mucosa, root coverage, peri-implant soft tissue, gingival fibroblasts, electrospinning and three-dimensional bioprinting, polymer-based drug delivery, and stimuli-responsive or smart polymers. Consistent with the narrative format, selection prioritized mechanistic clarity, representative preclinical work and the highest available level of clinical evidence for each intervention class rather than exhaustive coverage. The searches covered literature published up to and including April 2026, which is the publication date of the most recent studies included. No formal risk-of-bias or certainty-of-evidence assessment was undertaken, consistent with the narrative design; the strength of the evidence was instead characterized descriptively for each material and indication throughout the review.

## 2. Biological and Material Requirements for Polymer-Based Soft Tissue Regeneration

**Figure 2:**
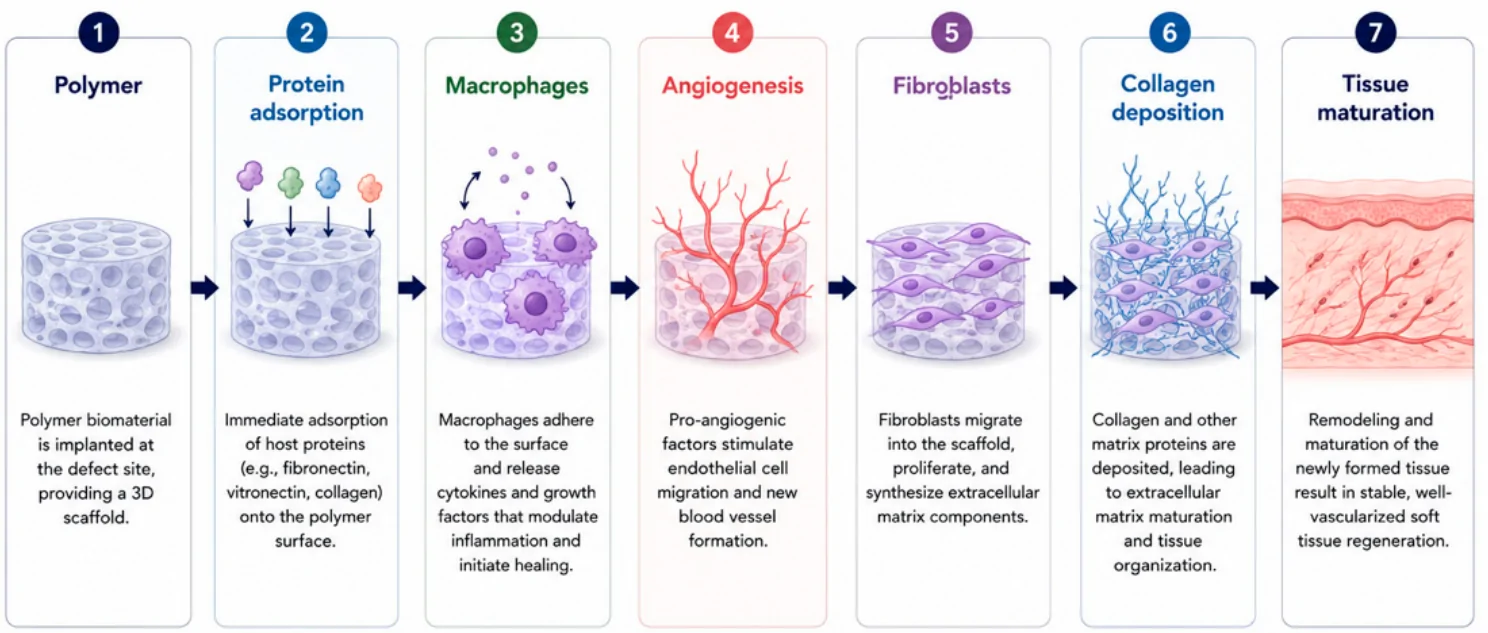
Biological mechanism of polymer-based biomaterials in soft tissue regeneration. The sequence of events following scaffold implantation is shown as seven consecutive stages, read left to right. (1) The polymer biomaterial is placed at the defect site and provides a three-dimensional porous scaffold. (2) Host proteins, including fibronectin, vitronectin and collagen, are adsorbed onto the polymer surface within minutes of implantation; this adsorbed protein layer, rather than the polymer itself, is what adherent cells first encounter. (3) Macrophages adhere to the conditioned surface and release cytokines and growth factors that modulate inflammation and initiate healing. (4) Pro-angiogenic factors stimulate endothelial cell migration and the formation of new blood vessels within the construct. (5) Fibroblasts migrate into the scaffold, proliferate and begin to synthesize extracellular matrix components. (6) Collagen and other matrix proteins are deposited, leading to matrix maturation and tissue organization. (7) Remodeling and maturation of the newly formed tissue result in stable, well-vascularized soft tissue regeneration. The polymer functions as a transient scaffold that orchestrates this cascade rather than as a permanent implant, and its degradation should be synchronized with stages 5 to 7.

Successful soft tissue augmentation depends on surgical technique and biological interactions between implanted biomaterials and host tissues. Modern regenerative biomaterials create a temporary extracellular matrix, supporting cell movement, controlling inflammation and facilitating soft tissue regeneration. Understanding the biological requirements for successful soft tissue regeneration is essential for designing and using polymer-based biomaterials. The healing of oral soft tissues involves many phases, including hemostasis, inflammation, proliferation, ECM deposition, angiogenesis, and tissue remodeling. Each stage is regulated by interactions among various cells and proteins. Consequently, the ideal polymeric scaffold should provide support for these processes while undergoing gradual degradation as the newly formed connective tissue matures [22,23,24,25,26,27] (Figure 2).

Regenerative dentistry has evolved, with biomaterials moving from passive tissue substitutes to bioactive platforms that modulate cellular behavior and facilitate tissue regeneration. This evolution is largely due to advances in polymer science, enabling precise control over the chemical, structural, mechanical, degradation and biological properties of scaffold materials. Biopolymers can be engineered to actively influence wound healing through interactions with host cells and the extracellular matrix [28,29].

To promote soft tissue regeneration around implants, polymeric biomaterials must meet specific biological and mechanical needs. The oral cavity is a demanding environment due to continuous microbial challenge, mechanical loading, exposure to saliva and rapid epithelial turnover. The success of polymeric scaffolds depends on their chemical composition and multiple structural and physicochemical characteristics that determine their interaction with the host tissues [30,31,32,33].

### 2.1. Hemostasis and Early Inflammatory Response

Immediately following surgical intervention, the formation of a stable blood clot provides the initial provisional matrix for wound healing. The fibrin network not only stabilizes the wound but also functions as a reservoir for platelets, leukocytes, and numerous bioactive molecules, including PDGF, TGF-β, VEGF, and FGFs. These signaling molecules initiate cellular recruitment and orchestrate subsequent healing events [34,35].

The implanted biomaterial quickly reacts with blood components through protein adsorption. This influences how cells respond, including the activation of macrophages, the spread of inflammatory signals and how the tissue integrates. The adsorption patterns of proteins are affected by surface chemistry, hydrophilicity, roughness, charge distribution and degradation products, which affect biological performance [36,37].

Although acute inflammation is essential for optimal wound healing, protracted or excessive inflammatory responses may impede angiogenesis, delay fibroblast migration, promote fibrosis, and compromise tissue regeneration. Consequently, contemporary polymeric biomaterials should not merely attempt to avoid inflammation; rather, they should promote a regulated immune response that fosters regenerative healing processes over destructive ones [38,39,40].

### 2.2. Immunomodulation and Macrophage Polarization

Increasing evidence indicates that macrophages represent central regulators of biomaterial-mediated tissue regeneration. Subsequent to implantation, circulating monocytes infiltrate the surgical site and differentiate into macrophages, which may exhibit distinct functional phenotypes depending on local environmental signals [41].

Classically activated M1 macrophages predominate during the early inflammatory phase and participate in microbial defense through TNF-α, IL-1β, and reactive oxygen species. As healing unfolds, successful tissue regeneration necessitates a gradual transition toward alternatively activated M2 macrophages, which secrete anti-inflammatory cytokines, stimulate angiogenesis, promote fibroblast proliferation, and facilitate ECM deposition [42,43].

The ability of polymeric biomaterials to influence immune response is crucial for successful regeneration. Natural polymers like collagen and hyaluronic acid have been shown to encourage a pro-regenerative immune environment. Synthetic polymers may need surface modification or biofunctionalization to achieve similar effects. As a result, immune compatibility has become a key design criterion for next-generation polymeric scaffolds [44,45,46,47].

### 2.3. Angiogenesis

The rapid establishment of a functional vascular network is indispensable for successful soft tissue regeneration. Newly formed blood vessels supply oxygen, nutrients, immune cells, and signaling molecules while eliminating metabolic waste. Insufficient vascularization is a primary cause of graft necrosis, delayed wound healing, and compromised tissue integration [48,49,50]. Polymer-based biomaterials should therefore facilitate endothelial cell migration, capillary sprouting, and neovascularization through appropriate scaffold architecture, interconnected porosity, and controlled delivery of angiogenic growth factors. Natural polymers, particularly collagen-based matrices and hyaluronic acid derivatives, possess intrinsic pro-angiogenic properties; synthetic polymers often require incorporation of bioactive molecules to stimulate vascular ingrowth [51,52,53].

### 2.4. Fibroblast Migration and Extracellular Matrix Formation

Fibroblasts have been identified as the principal effector cells responsible for regeneration of periodontal connective tissues. Subsequent to migration into the wound, fibroblasts proliferate and synthesize collagen types I and III, fibronectin, elastin, and proteoglycans, facilitating restoration of tissue integrity and mechanical strength during wound maturation. The ideal scaffold provides an appropriate three-dimensional microenvironment supporting fibroblast adhesion, migration, proliferation, and differentiation [54,55,56]. Scaffold architecture—pore size, fiber orientation, mechanical stiffness, and degradation kinetics—exerts a substantial influence on fibroblast behavior and thus on regenerated connective tissue quality. Excessive density may impede cellular infiltration, while overly porous structures may lack the mechanical stability to preserve tissue volume during healing [57,58].

### 2.5. Epithelial Integration and Soft Tissue Seal

The formation of a stable epithelial barrier is essential for protecting underlying connective tissues from microbial invasion, both around natural teeth and dental implants. While epithelial migration is imperative for wound closure, excessive epithelial proliferation may impede connective tissue regeneration and compromise mature soft tissue architecture [59,60,61]. Polymeric biomaterials should therefore promote balanced epithelial healing while supporting connective tissue maturation. The establishment of a stable peri-implant mucosal seal is of particular importance around dental implants; the absence of the periodontal ligament and differences in connective tissue fiber orientation render peri-implant tissues more susceptible to bacterial penetration and inflammatory breakdown [62,63].

The ideal polymer-based scaffold for soft tissue regeneration around implants should meet several criteria: It must be biocompatible, nonimmunogenic, mechanically stable during healing, and supportive of vascularization and fibroblast infiltration; it must modulate inflammation, promote ECM formation, maintain tissue volume, and undergo biodegradation while facilitating integration with surrounding tissues [64,65].

### 2.6. Natural and Synthetic Polymers

Polymeric biomaterials utilized in the regeneration of oral soft tissue can be broadly classified into two categories: naturally derived and synthetic polymers. Each group exhibits distinct advantages and limitations, and their selection is contingent upon the intended biological function and clinical application [64,65,66,67,68] (Table 1).

Natural polymers are obtained from biological sources and generally exhibit excellent biocompatibility because their molecular architecture closely resembles components of the native extracellular matrix. Examples include collagen, gelatin, hyaluronic acid, fibrin, chitosan, alginate, silk fibroin, and decellularized extracellular matrix-derived materials. These polymers inherently support cellular adhesion, migration, and differentiation while containing biochemical motifs recognized by integrins and other cell surface receptors. Their inherent bioactivity has been demonstrated to promote angiogenesis, collagen synthesis, and wound healing without necessitating extensive surface modification [207,208,209].

However, naturally derived polymers have limitations. The mechanical strength of these materials is comparatively low. Degradation rates often prove challenging to control; batch-to-batch variability can adversely affect reproducibility, and their biological origin introduces potential concerns regarding immunogenicity, disease transmission, and manufacturing standardization. Additionally, the process of rapid enzymatic degradation may diminish these cells’ capacity to regulate soft tissue volume during the prolonged recovery period [210,211,212,213] (Table 2).

Synthetic polymers offer greater flexibility in material design because their chemical composition and molecular architecture can be precisely controlled during manufacturing. A comprehensive investigation of synthetic polymers has revealed a range of materials that have been the focus of research, including PCL, PLA, PGA, PLGA, PEG, polyurethane, and PVA. These materials demonstrate predictable degradation behavior, superior mechanical stability, and excellent reproducibility, enabling fabrication of complex scaffold architectures through additive manufacturing, electrospinning, or three-dimensional printing [223,224,225,226].

The primary constraint on the utilization of synthetic polymers is their comparatively restricted biological activity. In contrast to naturally derived polymers, most synthetic materials lack intrinsic cell-recognition sequences. Consequently, biofunctionalization with extracellular matrix proteins, peptides, growth factors, or other bioactive molecules is required to enhance cellular attachment and tissue integration [227,228,229] (Table 3).

Consequently, there has been an increasing focus on hybrid polymeric systems that combine naturally derived and synthetic polymers. These systems leverage the biological advantages of the former while benefiting from the structural stability and tunable properties of the latter.

### 2.7. Controlled Biodegradation

Controlled biodegradation is a pivotal design parameter for regenerative scaffolds. In contrast to permanent implant materials, polymeric matrices utilized for soft tissue augmentation are designed to undergo a gradual dissolution process as newly formed connective tissue assumes its mechanical function. Consequently, the degradation of the scaffold should closely mirror the rate of tissue regeneration [230,231,232,233].

Natural polymers are subject to degradation via the action of enzymes, including collagenases, hyaluronidases, lysozyme, and matrix metalloproteinases, which are produced during the process of physiological wound healing. The degradation rates of these proteins can vary significantly depending on factors such as tissue enzyme activity and the presence of local inflammatory conditions [234,235].

It is crucial that an ideal regenerative scaffold exhibit degradation kinetics that are synchronized with connective tissue maturation. Excessively rapid degradation may result in premature collapse of the scaffold before sufficient connective tissue has formed, whereas prolonged persistence may interfere with tissue remodeling or promote chronic foreign body reactions. Furthermore, the degradation products of such a scaffold must be non-toxic, biocompatible, and readily metabolized by the host [236,237,238].

Biomaterial engineering has recently shifted focus from studying degradation mechanisms to controlling them through crosslinking density, polymer composition, molecular weight, and scaffold architecture [239,240,241,242,243,244].

### 2.8. Porosity and Microarchitecture

The structure of scaffolding plays a crucial role in tissue regeneration. Biomaterial performance is influenced by pore size and interconnectivity [245,246].

The interconnected porous structures allow various cell types to migrate throughout the scaffold, which is critical for neovascularization. Poor porosity can impede cellular penetration and prolong tissue integration, while excessively large pores have the potential to compromise mechanical integrity and reduce volume stability [247,248].

Substrate surface topography affects protein adsorption, adhesion and mechanotransduction, which regulate cellular differentiation. Contemporary fabrication techniques allow architecture manipulation, enhancing performance and stability [249,250,251].

### 2.9. Mechanical Properties, Stiffness, and Space Maintenance

The material’s mechanical properties significantly influence its surgical handling and biological behavior. The scaffold’s stiffness impacts resistance to deformation and cellular responses. Fibroblasts, macrophages and endothelial cells sense the mechanical characteristics of their environment and modify their phenotype accordingly [252,253,254,255].

Successful regeneration is predicated on preservation of adequate space for tissue ingrowth throughout healing. Premature scaffold collapse may impair vascularization, reduce connective tissue volume, and compromise clinical outcomes; conversely, excessive stiffness may interfere with physiological remodeling and increase foreign body reactions [256,257,258,259,260]. Mechanical properties should closely match recipient-site requirements—tensile strength, elasticity, compressive resistance, and structural integrity influence surgical handling, cellular behavior, and long-term tissue maturation. The capacity to customize these mechanical characteristics is a pivotal advantage of polymer-based biomaterials over traditional biological grafts [261,262,263,264].

Materials exhibiting insufficient stiffness may collapse under soft-tissue pressure, reducing space maintenance and compromising tissue volume. Conversely, excessively rigid scaffolds have been shown to impair physiological tissue remodeling, limit vascular ingrowth, and increase foreign body reactions. Consequently, polymers designed for oral soft tissue regeneration must exhibit mechanical properties that strike a balance between structural stability and biological flexibility [265,266].

The mechanical behavior of these materials can be further tailored through polymer blending, fiber reinforcement, multilayer scaffold design, or controlled crosslinking, allowing optimization for different clinical indications, ranging from gingival recession coverage to peri-implant phenotype enhancement [267,268,269] (Table 4).

### 2.10. Crosslinking

Crosslinking has been identified as a highly effective strategy for modifying the physical and biological properties of polymeric biomaterials [270,271]. Crosslinking, which is defined as the formation of covalent or non-covalent bonds between polymer chains, has been shown to result in several advantageous effects, including an increase in mechanical strength, an improvement in dimensional stability, a reduction in swelling, and a prolongation of degradation [272,273].

Crosslinking can be achieved through the use of chemical agents, enzymatic reactions, ultraviolet irradiation, or physical methods such as dehydrothermal treatment. The degree of crosslinking exerts a direct influence on the physical characteristics of the scaffold, including its stiffness, degradation rate, permeability, and the extent of cellular interactions within the construct [274,275].

While increased crosslinking has been shown to enhance structural stability, excessive crosslinking has been demonstrated to reduce pore size, impair cellular infiltration, decrease biodegradability, and limit vascularization. Consequently, achieving an optimal balance between mechanical durability and biological performance becomes paramount in crosslinking density optimization [276,277,278].

### 2.11. Bioactivity

The most significant development in polymer science is the transition to bioactive biomaterials. Scaffolds are now designed to regulate cellular behavior [279,280]. The incorporation of bioactive molecules and systems for controlled drug delivery has been demonstrated [281]. These modifications can enable polymeric scaffolds to modulate macrophage polarization, fibroblast activity, collagen deposition, and angiogenesis. At the same time, they have been shown to reduce bacterial colonization and inflammation [282,283].

Recent advancements have also introduced stimuli-responsive (“smart”) polymers capable of altering their physical or chemical properties in response to temperature, pH, enzymatic activity, or mechanical stress [284].

The utilization of such biomaterials facilitates the precise modulation of the release of therapeutic molecules, which can be synchronized with the various phases of wound healing. These biomaterials have the potential to represent a new generation of personalized regenerative therapies [285,286] (Table 5).

### 2.12. Biological–Polymer–Functional Agent Interactions

The biological requirements outlined above are not satisfied by bulk material properties alone. Whether a scaffold supports haemostasis, resolves inflammation, permits vascular ingrowth, sustains fibroblast colonization and establishes an epithelial seal depends on the molecular vocabulary the material presents to resident cells. In natural polymers this vocabulary is intrinsic: it consists of defined amino acid or saccharide domains that are recognized by specific cellular receptors. In synthetic polymers it is largely absent and must be introduced deliberately through biofunctionalization. Considering the biological–polymer–functional agent relationship therefore clarifies why chemically comparable scaffolds can elicit markedly different tissue responses and provides a rational basis for selecting or engineering a material for a defined biological objective.

Cell adhesion illustrates the principle most directly. The Arg-Gly-Asp (RGD) tripeptide, first identified in fibronectin, is recognized by several integrin heterodimers and remains the most widely applied adhesive motif in polymer functionalization; adhesion, spreading and migration depend not only on its presence but on its surface density, spatial arrangement and mode of tethering [287]. Fibrillar collagen presents a distinct high-affinity sequence, GFOGER, which is recognized by the collagen-binding integrins α1β1 and α2β1 and supports integrin-dependent adhesion in the native triple-helical conformation [288]. Hyaluronic acid signals principally through CD44 and RHAMM, and its biological effect is molecular-weight-dependent, with high-molecular-weight chains favoring an anti-inflammatory response while fragments generated during degradation act as pro-inflammatory and pro-migratory cues [289]. Sulfated domains, whether provided by heparin or by heparin-mimetic sequences, act through electrostatic affinity for the heparin-binding regions of VEGF, FGF-2 and SDF-1α, allowing a scaffold to sequester, protect and release growth factors in response to cellular demand rather than by passive diffusion [290].

Two consequences follow for material selection. First, the biological performance of a natural polymer is a property of its preserved functional domains, which means that processing steps that disrupt triple-helical conformation or remove glycosaminoglycan side chains may reduce bioactivity even when bulk architecture is retained. Second, the biological inertness of synthetic polymers is not a fixed limitation but an unoccupied design space: PEG in particular resists non-specific protein adsorption, which makes it a poor substrate in the unmodified state, and an unusually controllable one once defined ligands are introduced. Table 6 summarizes the principal functional domains exploited in periodontal and peri-implant soft tissue regeneration, the cellular partner each engages, the biological requirement it addresses, and the sections of this review in which the corresponding materials are discussed in detail.

### 2.13. Design Principles and Characteristics of an Ideal Polymeric Scaffold

These physicochemical properties determine the regenerative potential of polymer-based biomaterials [291,292,293,294]. These characteristics should be regarded as interconnected design variables that regulate the host biological response [295,296].

From a biological perspective, the ideal polymer-based scaffold for periodontal and peri-implant soft tissue regeneration should fulfill several fundamental requirements—biocompatibility, non-immunogenicity, mechanical stability during early healing, and capacity to support rapid vascularization and fibroblast infiltration. Concurrently, it should modulate rather than suppress the inflammatory response, promote ECM formation, maintain tissue volume, and undergo predictable biodegradation synchronized with connective tissue maturation. Finally, the scaffold should facilitate integration with surrounding tissues while serving as a platform for future biofunctionalization [297,298,299].

A comprehensive understanding of these principles provides the scientific foundation for interpreting the biological performance and clinical behavior of currently available polymeric biomaterials [300,301]. The ensuing sections offer a comprehensive review of individual classes of natural and synthetic polymers within the broader context of fundamental material science concepts. These sections also explore the applications of polymers in the field of periodontal and peri-implant soft tissue regeneration [302,303].

## 3. Natural Polymer-Based Biomaterials

Natural polymers are the most studied type of biomaterial used to aid the regeneration of soft tissue around teeth and implants [304]. These materials are similar to the natural ECM and are biocompatible [305]. They also create a suitable environment for cells to adhere, migrate, proliferate and remodel tissue [306]. Unlike most synthetic polymers, natural biomaterials have bioactive motifs that interact with cells [306].

The properties of natural polymers are determined by their composition and origin [307]. Most naturally derived biomaterials degrade during the process, generating non-toxic by-products while taking part in wound healing. These biomaterials should not be regarded as passive scaffolds [308]. However, challenges remain in developing chemically modified and hybrid polymer systems [309]. Among the natural polymers currently available, collagen-based matrices remain the most widely investigated materials in periodontal plastic surgery. In contrast, hyaluronic acid, gelatin, fibrin, extracellular matrix-derived scaffolds, chitosan, and alginate have demonstrated promising regenerative potential in both experimental and clinical studies [310,311].

### 3.1. Collagen

Collagen is the most abundant structural protein within the extracellular matrix of connective tissues and represents the cornerstone of contemporary polymer-based biomaterials for oral soft tissue regeneration [69]. It has been determined that the dry weight of connective tissue is composed of 70–80% collagen, with type I collagen being the predominant component of gingival connective tissue. Its distinctive fibrillar architecture provides both mechanical support and biological signals that regulate cell adhesion, migration, proliferation, and extracellular matrix remodeling [70].

Collagen biomaterials are mainly made from pig, cow or horse sources. The material is purified and processed to preserve its natural structure and minimize any possible immune reaction [71]. Commercial collagen matrices are typically composed of type I and III collagen arranged into 3-D porous structures to closely resemble the natural tissue environment [72]. This helps certain cells to attach and migrate, and its porous structure helps blood vessels to grow and the matrix to form [73]. Its breakdown products also help regulate wound healing by attracting certain cell types [74]. Collagen is a key protein in tooth-supporting connective tissues, so it usually integrates well with the body and causes little inflammation [75].

These biomatrices are being studied as an alternative to autogenous connective tissue grafts in several clinical procedures [76]. While randomized clinical trials have shown improvements in soft tissue thickness and keratinized tissue width, complete replacement of connective tissue grafts has not yet been achieved [77]. Collagen matrices have been shown to provide favorable patient-reported outcomes and reduced postoperative morbidity, but autogenous connective tissue grafts have been shown to be superior in demanding clinical situations [78].

Recent developments include multilayer collagen scaffolds, biofunctionalized collagen matrices, and collagen combined with growth factors, hyaluronic acid, or platelet concentrates to enhance angiogenesis and prolong scaffold stability [79].

### 3.2. Hyaluronic Acid

Hyaluronic acid (HA) is a naturally occurring non-sulfated glycosaminoglycan composed of repeating disaccharide units of glucuronic acid and N-acetylglucosamine [88]. As a significant component of the extracellular matrix, hyaluronan plays a crucial role in various biological processes, including tissue hydration, viscoelasticity, cell migration, angiogenesis, and modulation of inflammation [89].

A distinctive trait of HA is its molecular-weight-dependent activity [90]. High-molecular-weight HA has been shown to exhibit anti-inflammatory and immunomodulatory effects, while low-molecular-weight degradation fragments have been observed to stimulate angiogenesis and early wound healing through the activation of inflammatory signaling pathways [91]. This dual biological behavior enables HA to regulate different stages of tissue repair [92].

In the domain of regenerative dentistry, HA functions in two distinct capacities: first, as a hydrogel, and second, as a biologically active signaling molecule [93]. Crosslinked HA formulations have been shown to exhibit prolonged residence time and enhanced mechanical stability in comparison with native HA, while concurrently maintaining optimal biocompatibility [94]. A series of experimental studies have demonstrated that hyaluronic acid (HA) exerts a stimulatory effect on various biological processes, including fibroblast proliferation, collagen synthesis, epithelial migration, and neovascularization [95]. Concurrently, HA has been observed to attenuate oxidative stress and mitigate excessive inflammatory responses [96].

The clinical applications of this material include its use as an adjunct during root coverage procedures, the treatment of gingival recession, periodontal regeneration, peri-implant soft tissue augmentation, and enhancement of wound healing following periodontal surgery [97]. While the use of HA alone generally precludes its use in connective tissue grafts for procedures requiring significant volume augmentation, its use as an adjunctive regenerative material may improve early healing and reduce postoperative discomfort [98].

### 3.3. Gelatin

Gelatin is a denatured derivative of collagen that is obtained through the controlled hydrolysis of collagen fibers [80]. Despite the absence of a highly organized fibrillar structure characteristic of native collagen, gelatin possesses a wealth of bioactive amino acid sequences that promote cell adhesion and interaction with the extracellular matrix [81].

In comparison with collagen, gelatin demonstrates lower immunogenicity, greater processing flexibility, and improved solubility, rendering it a suitable material for the fabrication of hydrogels, injectable scaffolds, microspheres, and controlled drug-delivery systems [82]. The physicochemical properties of the material can be modified through a process known as crosslinking, which allows for the adjustment of degradation kinetics and mechanical performance [83].

Gelatin-based biomaterials have garnered significant attention as carriers for growth factors, antimicrobial agents, stem cells, and extracellular vesicles due to their substantial water content and exceptional loading capacity [84]. A substantial body of experimental research has demonstrated that gelatin-based scaffolds can promote fibroblast proliferation, accelerate angiogenesis, and enhance collagen deposition [85,86]. Despite the paucity of clinical evidence in the domain of periodontal plastic surgery, gelatin-based hydrogels are a rapidly expanding field of regenerative biomaterial research [87].

### 3.4. Fibrin

Fibrin is the principal structural protein of the provisional wound matrix formed during blood coagulation [115,116]. In contrast to collagen matrices, fibrin scaffolds have been shown to play a proactive role in the initial phases of wound healing by providing a provisional structural framework that facilitates platelet aggregation, the migration of inflammatory cells, the migration of fibroblasts, and the process of angiogenesis [117].

Autologous platelet concentrates, including platelet-rich fibrin (PRF), advanced PRF (A-PRF), injectable PRF (i-PRF), and concentrated growth factor (CGF) preparations, represent the most widely used fibrin-based biomaterials in contemporary regenerative dentistry [118]. These materials contain fibrin networks enriched with platelets, leukocytes, cytokines, and multiple growth factors, including PDGF, VEGF, TGF-β, IGF, and EGF [119].

The predominant advantage of fibrin is attributable to its intrinsic biological activity rather than its mechanical properties [120]. Given its rapid degradation rate, fibrin is incapable of maintaining tissue volume independently [121]. Consequently, it is primarily utilized as an adjunctive biomaterial in combination with connective tissue grafts, collagen matrices, or bone substitutes [122]. The extant evidence suggests that fibrin-based materials may accelerate early wound healing and improve postoperative comfort; however, their ability to replace autogenous grafts remains limited [123,124].

### 3.5. Extracellular Matrix-Derived Scaffolds

Decellularized extracellular matrix (ECM) scaffolds have emerged as an advanced class of naturally derived biomaterials designed to preserve the complex biological architecture of native connective tissues [214,215]. Subsequent to the removal of cellular components, these matrices retain collagen fibers, elastin, glycosaminoglycans, basement membrane proteins, and numerous matrix-bound signaling molecules [216].

In contrast to the use of purified collagen matrices, ECM-derived scaffolds offer a more physiologically relevant microenvironment, capable of regulating cell behavior through a variety of biochemical and biomechanical signals [217]. A body of experimental evidence has demonstrated that the implantation of ECM scaffolds results in enhanced angiogenesis, favorable macrophage polarization, accelerated fibroblast migration, and improved tissue remodeling [218,219].

The present clinical applications encompass soft tissue augmentation around teeth and implants, management of mucosal defects, and reconstruction following oral surgery [220,221]. While the technology shows promise, its widespread clinical adoption remains constrained by manufacturing complexity, cost, and the need for additional long-term clinical evidence [222].

### 3.6. Chitosan

Chitosan is a linear polysaccharide that is obtained through deacetylation of chitin, which is primarily derived from crustacean shells or fungal cell walls [99]. Due to its cationic nature, chitosan manifests distinctive biological properties that are not evident in the majority of other naturally derived polymers [100].

Chitosan has been shown to possess intrinsic antimicrobial, hemostatic, anti-inflammatory, and wound-healing properties [222]. The positive surface charge of the material facilitates interactions with negatively charged bacterial membranes and components of the extracellular matrix, while supporting fibroblast adhesion and collagen deposition. In addition, it has been demonstrated that chitosan possesses the capacity to stimulate the polarization of macrophages toward a regenerative phenotype and to promote angiogenesis [101,102].

A notable benefit of chitosan is its versatility [103]. The processing of this material into various forms, including membranes, hydrogels, nanoparticles, electrospun nanofibers, injectable systems, and composite scaffolds, is a subject of ongoing research [104,105]. These characteristics render chitosan a particularly attractive multifunctional platform for local drug delivery and regenerative medicine applications [106].

Despite the encouraging preclinical evidence, the clinical applications of chitosan in periodontal plastic surgery remain limited, and a recommendation of routine clinical implementation will require further randomized clinical trials.

### 3.7. Alginate

Alginate is a naturally occurring anionic polysaccharide that is extracted for its gelation capacity and mild gelation characteristics [107]. These properties render alginate attractive for use in injectable scaffolds and controlled delivery systems. However, alginate, by virtue of its absence of intrinsic cell-adhesion motifs, generally exhibits suboptimal cellular adhesion and frequently necessitates modification with adhesive peptides or combination with bioactive polymers such as collagen, gelatin, or other cell-adhesion-modifying agents [108].

In the domain of oral tissue engineering, alginate has emerged as a subject of increasing investigation. It is utilized as a component of composite hydrogels, bioinks for three-dimensional bioprinting, and stem-cell delivery systems [109,110,111]. Although the scope and application of present-day clinical trials are constrained by current limitations, there is considerable promise in alginate-based biomaterials as a foundation for next-generation, personalized regenerative therapies [112,113,114].

## 4. Synthetic Polymer Systems

Synthetic polymers are a rapidly growing class of biomaterials in regenerative medicine. They can be custom-made and are easy to reproduce. Unlike natural polymers, synthetic materials can be engineered to have specific properties, such as molecular weight, degradation rate, strength, porosity, and surface chemistry. This makes them useful in tissue engineering, controlled drug delivery, and 3D scaffold fabrication [312].

Synthetic polymers have several advantages over biologically derived materials when it comes to regeneration of periodontal soft tissue. They can be produced using advanced technologies, including electrospinning, additive manufacturing, solvent casting, freeze-drying, and three-dimensional bioprinting, enabling precise control of their structure and behavior [313,314].

Synthetic polymers generally lack intrinsic biological activity. Most materials lack natural cell-binding elements, so biofunctionalization is necessary to enhance cellular attachment and tissue integration. Contemporary research focuses on hybrid biomaterials that combine the structural advantages of synthetic polymers with the biological activity of naturally derived matrices [315,316].

Among the numerous synthetic polymers investigated for oral soft tissue regeneration, polycaprolactone (PCL), polylactic acid (PLA), polyglycolic acid (PGA), poly(lactic-co-glycolic acid) (PLGA), polyethylene glycol (PEG), and polyurethane have received the greatest scientific attention [317,318,319].

### 4.1. Polylactic Acid (PLA)

Polylactic acid (PLA) is a biodegradable aliphatic polyester that is synthesized from lactic acid, which is obtained through the fermentation of renewable carbohydrate sources. Due to its excellent biocompatibility, favorable mechanical properties, and predictable hydrolytic degradation, poly(lactic acid) (PLA) has become one of the most widely investigated synthetic polymers in the field of regenerative medicine [125,126].

The degradation of PLA occurs through the hydrolysis of ester bonds, producing lactic acid. This acid subsequently enters normal metabolic pathways via the Krebs cycle. The degradation rate is contingent upon polymer crystallinity, molecular weight, stereochemistry and local physiological conditions. This permits modification according to the intended clinical application [127].

PLA is suitable for the manufacture of porous scaffolds capable of maintaining tissue volume during early wound healing due to its relatively high mechanical stiffness and structural stability. However, the surface of the material is hydrophobic, which has the potential to limit cellular adhesion and vascularization. These processes can be modified through plasma treatment, peptide functionalization, or by combination with natural polymers [128,129,130].

The primary focus of research on PLA has been on its use as a scaffold for fibroblast culture, drug delivery, guided tissue regeneration, and soft tissue engineering. Electrospun PLA nanofibers exhibit a structural similarity to the architecture of native extracellular matrix, as evidenced by numerous experimental studies that have demonstrated favorable cellular responses [131,132,133,134].

### 4.2. Polyglycolic Acid (PGA)

Polyglycolic acid (PGA) is among the first synthetic polymers to be utilized in clinical medicine as a biodegradable material. In contrast to PLA, PGA demonstrates heightened hydrophilic properties and expedited degradation, attributable to its diminished crystallinity and augmented water absorbency [135,136]. The rapid degradation of PGA renders it particularly well-suited for applications necessitating temporary structural support during the nascent phase of wound healing. However, this characteristic may also impose limitations on its capacity to preserve long-term tissue volume, particularly in procedures necessitating prolonged scaffold stability [137].

PGA has been extensively utilized in absorbable surgical sutures and has subsequently been incorporated into tissue-engineering scaffolds and composite biomaterials [138,139]. In the context of periodontal regeneration, PGA-based matrices have been shown to facilitate fibroblast attachment and the deposition of the extracellular matrix. However, due to their rapid degradation rate, these matrices frequently require the incorporation of slower-degrading polymers such as polylactic-co-glycolic acid (PLGA) or polycaprolactone (PCL) [140,141].

### 4.3. Polycaprolactone (PCL)

Polycaprolactone (PCL) has emerged as a leading polymer in the field of oral tissue engineering due to its unique combination of mechanical flexibility, prolonged degradation profile, and excellent processability [142].

PCL exhibits a relatively slow degradation rate, often requiring several months to years for complete resorption. The rate of degradation is influenced by the geometry of the scaffold and its molecular weight. The prolonged structural stability exhibited by PCL renders it particularly well-suited for applications necessitating the maintenance of tissue volume over an extended period [143].

PCL exhibits a high degree of compatibility with electrospinning technology, facilitating the fabrication of nanofibrous scaffolds that closely resemble the fibrillar organization of native connective tissue. These scaffolds have been shown to promote fibroblast migration, collagen deposition, and angiogenesis while providing optimal mechanical support [144,145,146].

The primary constraint imposed by PCL is its hydrophobic surface, which diminishes protein adsorption and cellular attachment. Consequently, numerous studies have investigated surface modification techniques, including plasma treatment, collagen coating, incorporation of hyaluronic acid, or biofunctionalization with adhesive peptides to improve biological performance [147].

Due to its exceptional mechanical properties, PCL has emerged as a preferred polymer for the three-dimensional printing of customized scaffolds intended for oral soft tissue regeneration [148,149].

### 4.4. Polyethylene Glycol (PEG)

Polyethylene glycol (PEG) is a hydrophilic synthetic polymer that is biocompatible, immunogenic and chemically versatile and is used as a hydrogel-forming polymer. This enables it to form 3D networks that resemble native soft tissues. It can regulate hydrogel stiffness, swelling behavior, degradation and permeability through the manipulation of polymer chain length and crosslinking density. These properties render PEG an ideal platform for controlled drug delivery, encapsulation of living cells and sustained release of growth factors [156,157].

PEG itself is biologically inert and therefore does not actively promote cellular adhesion. Consequently, PEG hydrogels are frequently functionalized with extracellular matrix proteins, integrin-binding peptides, or bioactive molecules to enhance tissue integration and regenerative potential [158,159]. Recent investigations have explored the use of injectable polyethylene glycol-based hydrogels for minimally invasive soft tissue regeneration around teeth and implants, particularly as carriers for stem cells and bioactive proteins [160,161].

### 4.5. Poly(lactic-co-glycolic Acid) (PLGA)

Poly(lactic-co-glycolic acid) (PLGA) is a copolymer composed of lactic acid and glycolic acid units that combines the favorable characteristics of both PLA and PGA. The degradation rate of PLGA is predominantly determined by the proportion of lactic to glycolic acid monomers. This property facilitates the fabrication of scaffolds that maintain structural stability throughout the designated regenerative period [150].

PLGA has demonstrated excellent potential as a controlled drug-delivery platform capable of sustained release of antibiotics, anti-inflammatory agents, growth factors, antimicrobial peptides, and signaling molecules directly within healing periodontal tissues [151,152]. PLGA scaffolds have been shown to promote fibroblast growth and collagen production, while offering good mechanical stability. However, as with other synthetic polyesters, surface modification is often needed to boost cell attachment and blood vessel growth [153].

Due to its regulatory approval and extensive clinical experience in other medical disciplines, PLGA remains one of the most promising synthetic polymers for future translation into periodontal and peri-implant regenerative therapies [154,155].

### 4.6. Polyurethane

Polyurethanes constitute a diverse family of synthetic polymers distinguished by their exceptional elasticity, toughness, and mechanical durability. The chemical composition of these materials can be extensively modified through the selection of different polyols and isocyanates, allowing the fabrication of materials ranging from flexible hydrogels to rigid structural scaffolds. These materials are particularly well-suited for soft tissue applications that are subject to repetitive mechanical loading [162,163].

Recent developments have focused on biodegradable polyurethane formulations incorporating hydrolysable segments that permit gradual degradation of the scaffold while maintaining mechanical integrity during the initial healing phase. Furthermore, polyurethane scaffolds have demonstrated a favorable degree of cellular compatibility when incorporated with collagen, gelatin, hyaluronic acid, or bioactive nanoparticles [164,165].

Despite the present limitations in clinical applications in periodontal plastic surgery, polyurethane represents a promising platform for the future development of customized soft tissue substitutes with mechanical characteristics that closely resemble native oral mucosa [166,167,168].

## 5. Tissue Engineering

The evolution of polymer science in dentistry has led to new bioengineered constructs. Advanced constructs can replicate native oral soft tissues. Tissue engineering uses biodegradable polymeric scaffolds, living cells, bioactive molecules and advanced manufacturing technologies. This approach can be used to regenerate tissues rather than replacing volume. In plastic surgery for periodontal and peri-implant conditions, it is a strategy for overcoming the limitations of autogenous grafting [320,321,322,323].

Successful regeneration in tissue engineering depends on three components: a biocompatible scaffold, biological signals and responsive cells. Polymer-based biomaterials provide a temporary extracellular matrix that supports cell attachment, migration, proliferation, differentiation and tissue remodeling while delivering bioactive molecules. As a result, polymeric scaffolds have become essential tools for the current field of oral soft tissue engineering [324,325,326,327].

### 5.1. Natural–Synthetic Polymer Composites and Hybrid Polymer Scaffolds

The limitations of individual natural and synthetic polymers have driven the development of composite and hybrid polymeric scaffolds that combine the biological advantages of natural materials with the mechanical and physicochemical strengths of synthetic ones [169,170,171,172]. Rather than relying on a single biomaterial, contemporary scaffold engineering favors multifunctional designs that provide structural support, regulate cellular behavior, deliver bioactive molecules, and promote regeneration simultaneously [173,174].

Natural polymers (collagen, hyaluronic acid, gelatin, fibrin) offer strong biological activity but poor mechanical stability and rapid degradation. Synthetic polymers (PCL, PLGA, PLA, PEG) offer structural integrity and tunable degradation but lack intrinsic bioactivity. Hybrid scaffolds combine both to offset these limitations. Collagen- or gelatin-coated synthetic polyesters are among the most studied systems: the synthetic component provides mechanical stability while the natural component supports fibroblast attachment, angiogenesis, and matrix deposition [175,176,177].

Injectable hydrogels have emerged as minimally invasive alternatives to conventional scaffold implantation. These materials undergo a sol-to-gel transition following injection, allowing complete adaptation to irregular tissue defects while minimizing surgical trauma. Hybrid injectable systems frequently integrate polyethylene glycol (PEG), hyaluronic acid, gelatin, collagen, or chitosan with synthetic crosslinking agents to attain optimal injectability, mechanical stability, and biodegradation. The high-water content of these materials closely resembles that of native connective tissue, thereby providing an optimal environment for cell survival and migration [178].

Beyond functioning as structural matrices, injectable hydrogels serve as delivery platforms for a variety of biological substances, including growth factors, antimicrobial agents, stem cells and extracellular vesicles, including exosomes. The controlled release of these biological mediators enables temporal regulation of wound healing, thereby supporting various stages of tissue regeneration, from the initial phase of inflammation to matrix remodeling [179,180].

Because native oral soft tissues have a hierarchical, multilayered architecture, scaffold design increasingly mimics this organization through multilayer and gradient constructs that vary composition, porosity, or stiffness to support distinct cellular processes at different depths and improve integration with host tissue [181,182].

Precise definition of individual defect anatomy, including through advanced imaging modalities, further supports the design of such biomimetic constructs [183]. Electrospinning enables fabrication of nanofibrous scaffolds resembling native collagen architecture, with tunable fiber diameter, alignment, and porosity that support fibroblast migration, angiogenesis, and connective tissue organization [184].

Composite electrospun scaffolds combining PCL, PLA, or PLGA with collagen, gelatin, or chitosan are particularly relevant for periodontal and peri-implant applications. Injectable hydrogels offer a minimally invasive alternative, conforming to irregular defects via sol-to-gel transition and serving as delivery vehicles for growth factors, antimicrobials, stem cells, and extracellular vesicles to support sequential stages of wound healing [185,186].

A significant trend in this field involves a transition from structurally optimized to biologically instructive scaffolds. These scaffolds are modified with adhesive peptides such as RGD, as well as growth factors including VEGF, FGF, PDGF, antimicrobial peptides, and anti-inflammatory agents. The purpose of these modifications is to actively direct various biological processes such as angiogenesis, macrophage polarization, and tissue maturation. Furthermore, there are systems designed for sequential, programmable release that mirror physiological healing [187,188].

Additive manufacturing, particularly 3D printing with composite bioinks (e.g., GelMA, collagen, alginate, PEG, PCL), allows patient-specific scaffold fabrication tailored to complex anatomical defects, with particular promise for peri-implant and mucogingival reconstruction [189,190,191].

Despite these advances, clinical translation remains limited by manufacturing complexity, sterilization and storage challenges, regulatory hurdles, cost, and a lack of robust clinical trial evidence. Future development is expected to focus on intelligent, multifunctional biomaterials integrating structural support, immunomodulation, antimicrobial protection, and programmable bioactive delivery—potentially guided by AI-assisted design—to produce personalized scaffolds that closely replicate native periodontal and peri-implant soft tissue behavior [192,193,194,195,196].

### 5.2. Stem Cell-Based Regenerative Strategies

The investigation of stem cells has been a primary focus in the domain of tissue engineering. These cells possess a remarkable ability to proliferate and differentiate into various lineages, in addition to secreting a multitude of factors that promote regeneration. Mesenchymal stem cells (MSCs) primarily demonstrate their preclinical or clinical significance through their non-stem/progenitor cell functions by producing extracellular vesicles, cytokines, and growth factors that modulate immune responses [328,329].

A number of stem cell populations have been the focus of research in the context of periodontal and peri-implant soft tissue regeneration. MSCs derived from bone marrow, adipose tissue, periodontal ligament, gingiva, dental pulp, and oral mucosa have demonstrated the ability to enhance angiogenesis, collagen synthesis, fibroblast proliferation, and extracellular matrix remodeling [330,331].

Among these, gingiva-derived mesenchymal stem cells (GMSCs) and periodontal ligament stem cells (PDLSCs) are of particular interest due to their accessibility, favorable immunomodulatory properties, and tissue-specific regenerative potential [332,333].

Polymeric scaffolds provide a three-dimensional microenvironment that protects transplanted cells, facilitates nutrient diffusion, and supports cellular organization during tissue formation. Mesenchymal stem cells derived from bone marrow, adipose tissue, periodontal ligament, and gingival connective tissue have demonstrated considerable regenerative potential when combined with biodegradable polymeric scaffolds [334,335].

The influence of scaffold characteristics, including but not limited to pore size, stiffness, degradation kinetics, and surface chemistry, on stem cell survival, proliferation, and lineage commitment is significant. Consequently, the optimization of scaffold architecture has become equally important as the selection of the cellular component itself [336,337].

Notwithstanding the encouraging experimental results, several challenges persist that constrain clinical translation, including cell harvesting, expansion protocols, regulatory approval, manufacturing costs, and standardization of therapeutic products. These limitations have thus given rise to a growing interest in cell-free regenerative approaches based on extracellular vesicles and secretome-derived therapies [338,339].

### 5.3. Extracellular Vesicle Delivery

Extracellular vesicles (EVs), including exosomes, have attracted increasing interest as mediators of intercellular communication. Exosomes transfer biological information molecules, such as DNA, proteins and microRNA, from releasing cells to recipient cells, exerting various biological effects. These particles have garnered attention due to their ability to preserve the regenerative characteristics of stem cells while circumventing the constraints often associated with cell transplantation [340,341,342].

Some researchers have applied exosome technology to the field of periodontal tissue regeneration. Exosomes derived from stem cells from human exfoliated deciduous teeth (SHEDs) have been reported to modulate the behavior of periodontal ligament cells, including their proliferation and matrix-forming activity [343].

Adipose-derived stem cells (ADSCs) have been shown to secrete exosome-rich conditioned medium, which has been investigated as a potential adjunctive therapy for experimental periodontitis in rats [344,345].

Polymeric matrices capable of gradually releasing extracellular vesicles have demonstrated enhanced angiogenesis, collagen synthesis, fibroblast proliferation, and immunomodulation in experimental models [346,347,348].

### 5.4. Growth Factor-Based Tissue Engineering

Growth factors represent a prominent class of therapeutic molecules that have been extensively investigated for incorporation into polymeric scaffolds in the context of periodontal regeneration [349,350,351,352]. Polymeric biomaterials have been shown to enable localized, sustained release of growth factors, including PDGF, VEGF, FGFs, TGF-β, BMPs, EGF, and IGF [353,354,355].

For soft tissue applications, the most relevant growth-factor-mediated effects include angiogenesis, fibroblast recruitment and proliferation, extracellular matrix deposition and the modulation of inflammation. Platelet-derived growth factor promotes fibroblast chemotaxis and proliferation and supports early matrix formation [356,357]. Fibroblast growth factor-2 (FGF-2) is a heparin-binding cytokine that stimulates angiogenesis and fibroblast activity, both of which are rate-limiting for soft tissue regeneration [358,359,360].

Growth factors acting primarily on mineralized tissues, including the bone morphogenetic proteins, are not considered further here, since the present review is restricted to soft tissue augmentation [361,362,363,364].

In comparison with conventional administration, sustained release from biodegradable polymers has been shown to protect growth factors from premature degradation, prolong biological activity, and maintain therapeutic concentrations during the critical early phases of healing [365,366,367,368].

### 5.5. Three-Dimensional Bioprinting

Three-dimensional (3D) printing has fundamentally expanded the possibilities of polymer-based tissue engineering by enabling the fabrication of patient-specific scaffolds with precisely controlled geometry, porosity, and internal architecture [369].

Hydrogels derived from gelatin methacrylate (GelMA), collagen, alginate, hyaluronic acid, polyethylene glycol (PEG), and composite polymer systems have emerged as the predominant bioinks for oral soft tissue engineering [370,371]. Three-dimensional bioprinting further enables spatial distribution of multiple cell populations, growth factors, and polymer compositions within a single construct [372,373,374].

Future applications may include individualized peri-implant soft tissue substitutes, customized ridge contour augmentation, and personalized mucogingival grafts fabricated directly from patient-derived digital datasets [375].

### 5.6. Electrospinning Technology

Electrospinning has emerged as a leading manufacturing technique for polymer-based soft tissue engineering due to its ability to produce nanofibrous scaffolds that closely resemble the ultrastructure of the native extracellular matrix [197,198]. The process of electrospinning has been demonstrated to be applicable to a wide range of polymers, encompassing both natural and synthetic materials [199,200,201].

Electrospun scaffolds have been demonstrated to support fibroblast migration, angiogenesis, collagen deposition, and extracellular matrix remodeling while concurrently functioning as platforms for controlled delivery of growth factors, antimicrobial agents, stem cells, extracellular vesicles, and other therapeutic molecules [202,203,204,205]. While electrospinning remains an experimental technology in the field of periodontology, it is regarded as one of the most promising manufacturing approaches for future polymer-based soft tissue substitutes [206].

### 5.7. Polymer-Based Drug Delivery

Polymer-based drug delivery systems (DDSs) have emerged as a promising strategy for enhancing periodontal and peri-implant soft tissue regeneration [376,377,378]. In contrast to conventional administration routes, localized polymeric delivery systems enable precise spatial and temporal control over drug release [379,380,381]. The release profile of therapeutic agents is primarily determined by the physicochemical properties of the polymer, including molecular weight, hydrophilicity, porosity, degradation kinetics, crosslinking density, and scaffold architecture [382].

Modern polymer engineering facilitates precise modulation of release kinetics, thereby enabling the delivery of therapeutic molecules according to the biological requirements of the various stages of wound healing [383]. Hydrogels, electrospun nanofibers, biodegradable microspheres, and nanoparticle-loaded polymer matrices have demonstrated prolonged antimicrobial activity while maintaining favorable cellular compatibility [384,385,386].

The successful regeneration of tissue is contingent upon precise regulation of inflammation, as opposed to the complete suppression of the immune response [387,388]. Polymeric scaffolds have therefore been investigated as delivery vehicles for anti-inflammatory agents, corticosteroids, non-steroidal anti-inflammatory drugs, cytokines, specialized pro-resolving mediators, and immunomodulatory molecules capable of influencing macrophage polarization [389,390].

Of particular significance is the capacity of immunomodulatory polymeric scaffolds to not only impede inflammation but also to actively promote the transition from pro-inflammatory M1 macrophages to regenerative M2 phenotypes [391]. This concept of immunoengineering has emerged as one of the most rapidly developing areas of biomaterial science and is expected to play a central role in future periodontal regeneration [392].

### 5.8. Smart and Stimuli-Responsive Drug Delivery Systems

One of the most significant advancements in polymer science is the development of smart biomaterials that exhibit the capacity to respond dynamically to changes in the local biological environment [393,394,395]. Within the oral cavity, inflammatory conditions are frequently associated with reduced pH, elevated protease activity, and increased concentrations of reactive oxygen species [396,397,398].

For instance, pH-sensitive hydrogels have the capacity to selectively release antimicrobial agents in acidic inflammatory environments, whereas enzyme-responsive polymers degrade preferentially in the presence of matrix metalloproteinases associated with active tissue destruction [399,400,401].

## 6. Clinical Applications

Polymer-based biomaterials have become increasingly incorporated into periodontal plastic surgery and peri-implant soft tissue management as alternatives or adjuncts to autogenous grafting procedures [402]. Although connective tissue grafts remain the reference standard for most soft tissue augmentation procedures, polymeric biomaterials offer important clinical advantages, including elimination of donor-site morbidity, reduced surgical time, improved patient comfort, and unlimited material availability. Their clinical effectiveness, however, varies considerably depending on the specific indication, the biological characteristics of the recipient site, and the physicochemical properties of the biomaterial [403].

**Figure 3:**
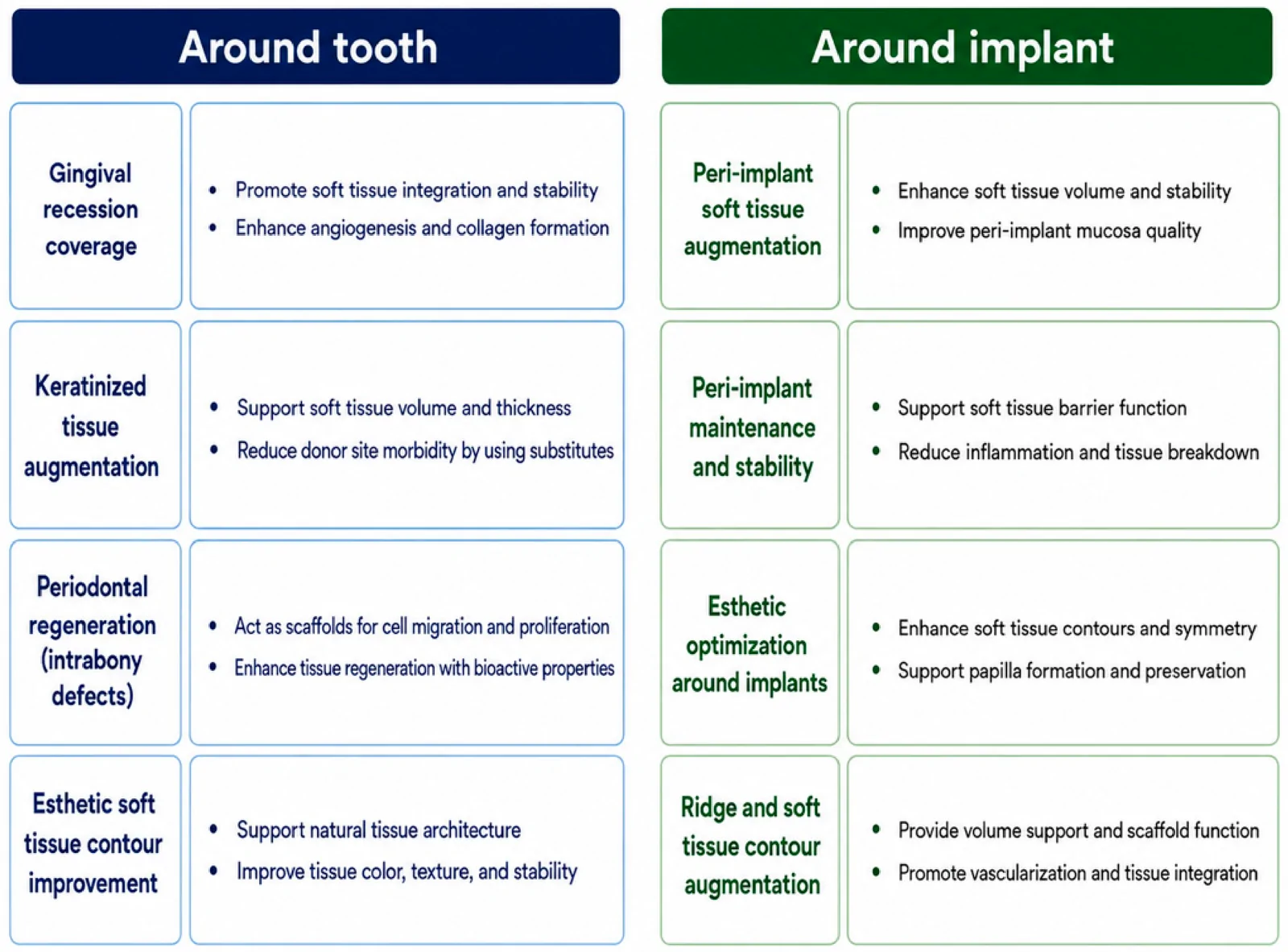
Clinical applications of polymer-based biomaterials, presented in two parallel columns. The left column (blue) covers indications around teeth: gingival recession coverage, keratinized tissue augmentation, periodontal regeneration in intrabony defects, and esthetic soft tissue contour improvement. The right column (green) covers indications around implants: peri-implant soft tissue augmentation, peri-implant maintenance and stability, esthetic optimization around implants, and ridge and soft tissue contour augmentation. For each indication the figure lists the principal contributions of polymer-based biomaterials to that clinical goal. The paired layout is intended to show where the two anatomical settings share a rationale and where they diverge; volume and thickness objectives predominate around implants, whereas coverage and attachment objectives predominate around teeth.

Current clinical applications extend beyond simple volume replacement and increasingly focus on biologically driven modulation of wound healing, phenotype enhancement, and long-term maintenance of periodontal and peri-implant tissue stability. The following sections summarize the principal indications for polymer-based biomaterials in contemporary periodontal and implant therapy (Figure 3 and Table 7).

### 6.1. Root Coverage Procedures

Treatment of gingival recession is the most extensively investigated indication for polymer-based biomaterials. Root coverage is among the most common periodontal plastic procedures, as recession can cause hypersensitivity, cervical caries, non-carious lesions, and esthetic concerns [408].

Autogenous connective tissue grafting with a coronally advanced flap remains the gold standard for complete, stable root coverage. Collagen matrices are the most studied polymeric alternative, and multiple randomized trials show they improve recession reduction, gingival thickness, and patient-reported outcomes while avoiding donor-site morbidity [409,410,411]. However, evidence consistently shows collagen substitutes achieve lower rates of complete root coverage and less long-term volumetric stability than connective tissue grafts, particularly in multiple recessions and thin phenotypes. Polymeric biomaterials are therefore best regarded as valuable alternatives when morbidity concerns, limited donor tissue, or patient preference preclude autogenous grafting—not as universal replacements [412,413,414].

Adjuncts such as hyaluronic acid and platelet-rich fibrin may further improve early healing, reduce discomfort, and enhance tissue maturation when combined with conventional root coverage techniques [415,416,417].

### 6.2. Keratinized Tissue Augmentation

The role of keratinized tissue around teeth and implants remains debated, but growing evidence suggests an adequate zone of keratinized mucosa facilitates plaque control, reduces discomfort during oral hygiene, and supports long-term periodontal and peri-implant stability [404,405].

Free gingival grafts remain the gold standard for increasing keratinized tissue width given their predictability and long-term stability. Collagen matrices have emerged as attractive alternatives, offering less postoperative pain and eliminating palatal harvesting [418,419].

Clinical trials show collagen matrices effectively increase keratinized tissue width, though typically less than free gingival grafts, with greater postoperative shrinkage and reduced volumetric stability. Patient satisfaction and quality of life, however, are consistently higher with collagen-based biomaterials [420,421,422].

### 6.3. Periodontal Phenotype Modification

Optimization of the periodontal phenotype has become a fundamental component of contemporary periodontal and restorative treatment planning. A thin, soft tissue phenotype is associated with increased susceptibility to gingival recession, marginal tissue instability, esthetic complications, and compromised outcomes following orthodontic movement or restorative procedures [423,424,425]. Polymer-based biomaterials are increasingly employed to increase soft tissue thickness and improve phenotype before orthodontic treatment, implant placement, or restorative rehabilitation [426,427,428].

Collagen matrices currently represent the most extensively investigated materials for phenotype enhancement, demonstrating clinically significant increases in soft tissue thickness with substantially reduced patient morbidity. Hyaluronic acid has also shown promising results as an adjunctive biomaterial by enhancing angiogenesis, collagen synthesis, and connective tissue maturation during healing [429,430].

### 6.4. Ridge Preservation and Ridge Contour Enhancement

Preservation of alveolar ridge contours after tooth extraction is essential for predictable esthetic and functional outcomes in implant therapy [431,432].

Polymeric biomaterials serve both as soft tissue substitutes covering extraction sockets and as adjunctive matrices supporting contour development after bone augmentation. Collagen matrices are frequently used for socket sealing, stabilizing the blood clot, protecting graft material, and facilitating epithelial healing without palatal harvesting [433,434]. Hybrid scaffolds combining collagen with synthetic polymers or bioactive hydrogels are increasingly investigated to enhance soft tissue volume maintenance and angiogenesis [431,435,436].

### 6.5. Peri-Implant Soft Tissue Augmentation

The optimization of peri-implant soft tissues has emerged as a pivotal objective in contemporary implant dentistry [437,438]. Conventionally, peri-implant soft tissue augmentation has relied on autogenous connective tissue grafts harvested from the palate [439,440]. Collagen matrices currently represent the most clinically investigated biomaterials for peri-implant soft tissue augmentation. A substantial body of research has demonstrated that randomized clinical trials have yielded notable increases in peri-implant mucosal thickness, accompanied by a concomitant reduction in postoperative pain when compared with connective tissue grafting [441,442].

Beyond collagen, biofunctionalized hydrogels, extracellular matrix-derived scaffolds, and hybrid polymeric constructs have demonstrated encouraging preclinical results by promoting angiogenesis, fibroblast proliferation, and connective tissue integration around implant abutments [443,444,445]. Given the increasing prevalence of peri-implant diseases and the growing emphasis on peri-implant phenotype optimization, polymer-based biomaterials are expected to play an increasingly important role in implant therapy over the coming decade [446].

### 6.6. Clinical Perspective

Current evidence suggests polymer-based biomaterials should not be viewed as universal substitutes for autogenous grafts but as indication-specific regenerative tools [447,448,449]. As biomaterial engineering evolves, future polymeric scaffolds incorporating controlled drug delivery, immunomodulatory molecules, bioactive peptides, extracellular vesicles, and patient-specific 3D architectures are expected to narrow the gap between synthetic substitutes and autogenous grafts [406,407] (Table 8 and Table 9).

### 6.7. Magnitude of Clinical Differences Compared with Autogenous Grafts

Statements that collagen matrices are the best-documented alternative to autogenous grafting are only informative if the size of the residual difference is stated. Across indications, the pattern is consistent: polymer-based substitutes reproduce much, but not all, of the effect of autogenous tissue, and the gap is largest where the clinical objective is tissue volume or keratinization rather than defect coverage.

In root coverage, a meta-analysis of randomized trials in multiple adjacent recessions found significantly lower recession reduction, complete root coverage and mean root coverage with xenogeneic collagen matrix than with connective tissue graft, while recession width, clinical attachment level and keratinized tissue width did not differ significantly between groups [409]. For peri-implant soft tissue thickness, a recent multicenter non-inferiority randomized trial reported a mean increase of 1.0 ± 0.75 mm with connective tissue graft against 0.66 ± 0.58 mm with a cross-linked volume-stable collagen matrix at 12 months [450]. In keratinized tissue augmentation, the difference is larger still, with autogenous free gingival grafting consistently producing greater width gain than collagen matrix, at the cost of greater morbidity and inferior color match.

Three qualifications follow. First, differences of a few tenths of a millimeter may be statistically significant without being clinically decisive, particularly where the baseline deficiency is modest. Second, patient-reported outcomes move in the opposite direction: substitutes shorten operating time, avoid a palatal donor site and reduce postoperative pain, so the comparison is a trade-off rather than a ranking [450]. Third, the heterogeneity of surgical technique, defect classification and follow-up across trials means pooled estimates should be read as approximate. Table 10 summarizes the principal quantitative comparisons.

## 7. Clinical Decision-Making

The selection of an appropriate polymer-based biomaterial for periodontal and peri-implant soft tissue augmentation necessitates the integration of biological principles, defect characteristics, patient factors, and expected outcomes. Currently, no single material exists that can adequately substitute for autogenous connective tissue grafts in all clinical contexts. As a result, treatment decisions should be driven by the specific indication rather than by the material itself. Substitutes should be chosen primarily in cases where there are donor-site morbidity concerns, limited tissue availability, patient preference, or systemic conditions that favor their use [452,453].

### 7.1. Defect Characteristics

The morphology of defects and the inherent biological complexity of materials under consideration are the primary factors that determine the selection of biomaterials. In isolated recessions with favorable anatomy, collagen matrices can provide satisfactory outcomes with substantially reduced morbidity; however, deep or multiple recessions and thin phenotypes still favor autogenous grafts for superior root coverage and stability. Additionally, extensive keratinized tissue deficiency—particularly in the mandibular anterior region and around implants—continues to favor free gingival grafts over collagen matrices. In the context of peri-implant reconstruction, the management of small-volume deficiencies can be addressed through the utilization of collagen-based substitutes. Conversely, cases involving severe buccal soft tissue collapse typically necessitate autogenous grafting [454,455,456,457,458,459,460,461].

### 7.2. Patient-Related Factors

Patient preference has emerged as a pivotal factor in treatment selection, as many patients are reluctant to undergo palatal harvesting due to the associated pain and prolonged healing time. A number of factors may also favor the use of less invasive polymer-based approaches, including impaired wound healing, systemic inflammatory disease, anticoagulant therapy, or limited surgical tolerance. Smoking, uncontrolled diabetes, and inadequate plaque control persist as significant risk factors irrespective of biomaterial selection, exerting a deleterious influence on the healing process following periodontal surgical interventions. The optimization of these factors should be prioritized prior to augmentation, whenever feasible [462,463,464,465,466].

### 7.3. Material Selection According to Clinical Objectives

The material selection should be guided by the primary therapeutic objective. Evidence supporting the use of naturally derived polymers (e.g., collagen, hyaluronic acid) in enhancing wound healing and reducing morbidity is substantial. Synthetic polymers and mechanically reinforced hybrid scaffolds offer advantages in circumstances necessitating prolonged structural support and volume maintenance due to their reduced degradation rates and enhanced dimensional stability. Hydrogels, electrospun nanofibers, and composite systems are particularly well-suited to applications necessitating the sustained, controlled release of therapeutic agents [467,468,469,470,471,472,473,474].

### 7.4. Evidence-Based Clinical Recommendations

The current body of clinical evidence suggests that collagen matrices should be considered as alternatives to connective tissue grafts, with the caveat that reduced morbidity is of higher importance than maximal volumetric gain. Hyaluronic acid is regarded as a biologically active adjunct rather than a substitute in and of itself. The use of platelet-derived fibrin matrices has been demonstrated to enhance early healing and comfort; however, the current body of evidence is insufficient to support their use as a replacement for autogenous grafts in major augmentation procedures. Synthetic polymers are predominantly in the preclinical stage, with the most significant current applications being in drug delivery and the development of next-generation scaffolds. Hybrid scaffolds represent a promising future direction, pending further randomized trials with long-term follow-up [475,476,477,478,479,480,481,482,483,484] (Table 11).

### 7.5. Proposed Clinical Algorithm

**Figure 4:**
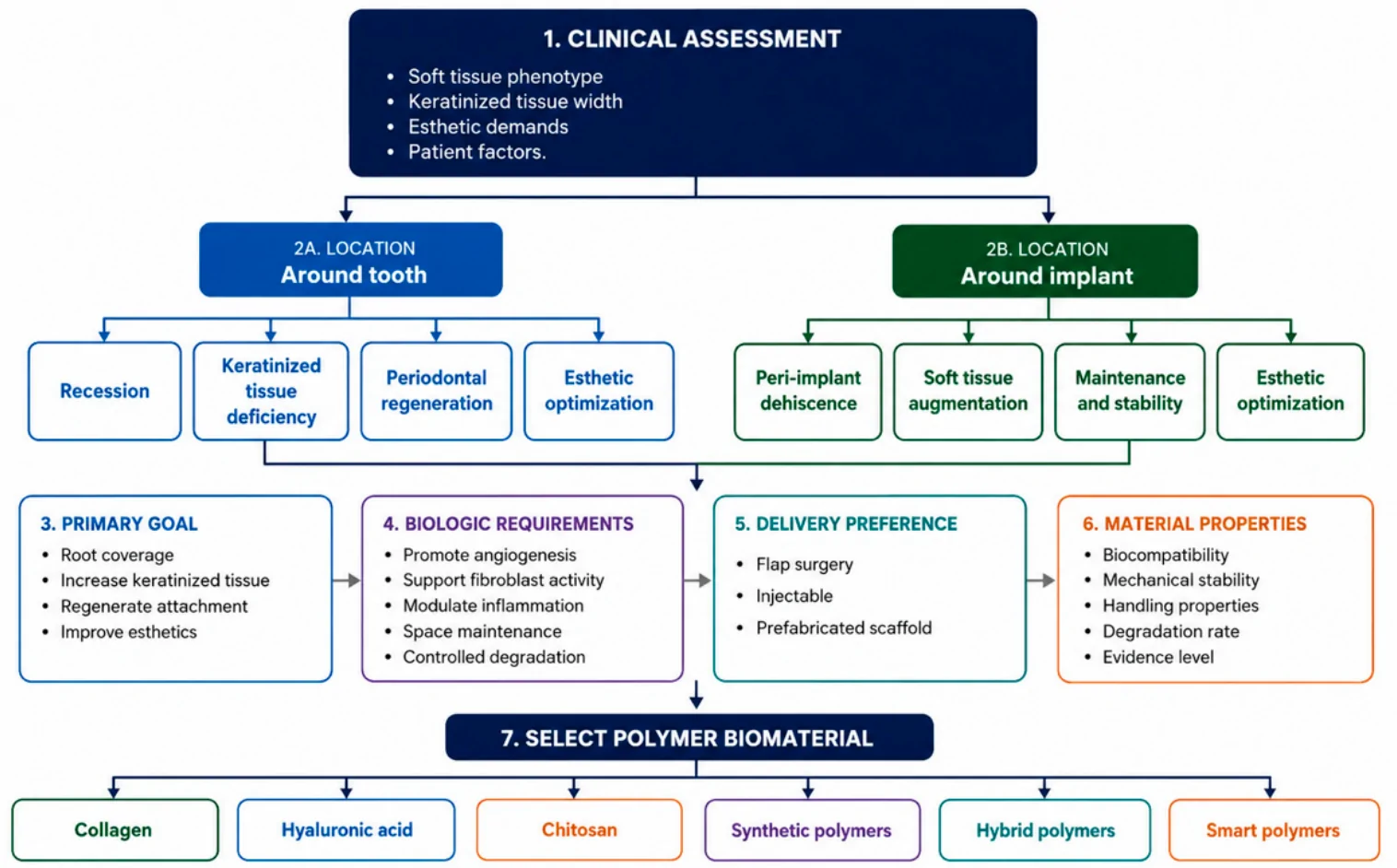
Decision algorithm for selecting a polymer-based biomaterial in periodontal and peri-implant soft tissue regeneration. The algorithm is read from top to bottom in seven steps. Step 1, clinical assessment, records soft tissue phenotype, keratinized tissue width, esthetic demands and patient factors. Step 2 separates the two anatomical settings: around tooth (2A, blue), with the subtypes recession, keratinized tissue deficiency, periodontal regeneration and esthetic optimization; and around implant (2B, green), with the subtypes peri-implant dehiscence, soft tissue augmentation, maintenance and stability, and esthetic optimization. Both branches converge on step 3, definition of the primary goal, followed by step 4, biologic requirements; step 5, delivery preference; and step 6, material properties, which include the level of supporting evidence. Step 7 is selection of the material, with the six candidate classes shown in the lower panel: collagen, hyaluronic acid, chitosan, synthetic polymers, hybrid polymers and smart polymers. The characteristics of each class, and the evidence supporting its use in each indication, are given in Table 1, Table 2, Table 3, Table 4, Table 5, Table 6 and Table 7. The algorithm is derived from the literature and from clinical reasoning and has not been prospectively validated.

The clinical decision-making process for polymer-based soft tissue augmentation should adhere to a structured sequence, commencing with a comprehensive patient assessment and clearly defined treatment objectives [494]. The clinician must first ascertain the primary indication for augmentation, including root coverage, phenotype modification, keratinized tissue augmentation, ridge contour enhancement, or peri-implant soft tissue reconstruction. Subsequently, a comprehensive evaluation of patient-specific factors must be conducted. These factors include systemic health, smoking status, esthetic demands, willingness to undergo donor-site surgery, and anticipated maintenance [495,496,497] (Figure 4).

The subsequent step involves the assessment of defect severity and the desired degree of tissue augmentation. In cases necessitating maximal tissue volume, long-term dimensional stability, or highly demanding esthetic outcomes, autogenous connective tissue grafting remains a viable treatment option. Conversely, moderate defects or situations prioritizing reduced surgical morbidity may be managed successfully using polymer-based biomaterials [498,499,500,501,502].

**Figure 5:**
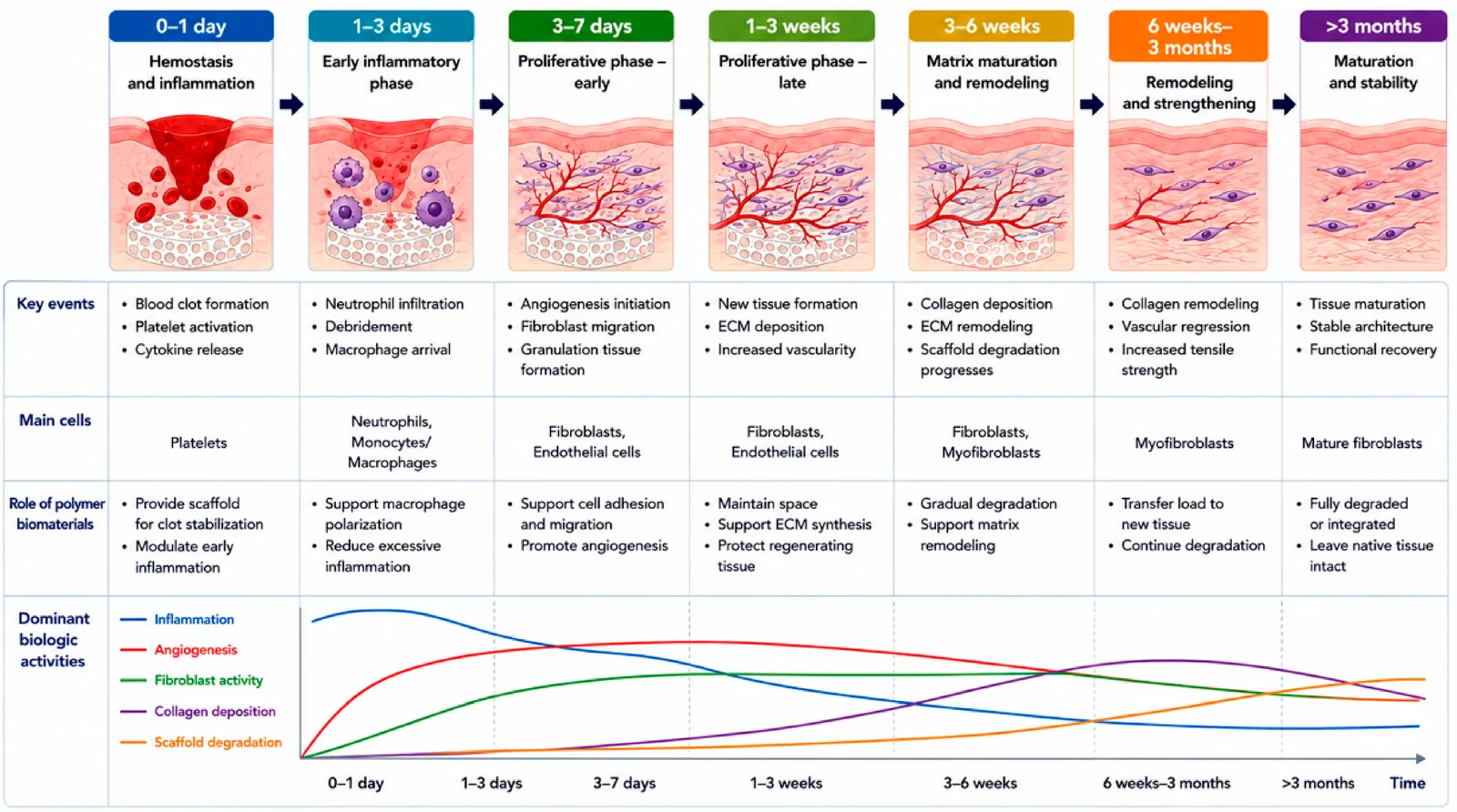
Timeline of soft tissue wound healing in the presence of a polymer-based biomaterial. Seven consecutive intervals are shown across the top, from hemostasis and inflammation (0–1 day) through the early and late proliferative phases, matrix maturation and remodeling, to maturation and stability beyond three months. The three central rows give, for each interval, the key biological events, the predominant cell populations (platelets; neutrophils, monocytes and macrophages; fibroblasts and endothelial cells; myofibroblasts; and mature fibroblasts), and the corresponding role of the polymer biomaterial, which shifts from clot stabilization and modulation of early inflammation, through support of cell adhesion, migration and angiogenesis, to space maintenance and finally to controlled degradation and load transfer to the new tissue. The lower panel plots the relative intensity over time of five dominant biological activities: inflammation (blue), angiogenesis (red), fibroblast activity (green), collagen deposition (purple) and scaffold degradation (orange). Curves are schematic and indicate relative rather than absolute magnitude; the intervals are indicative and vary with defect type, patient factors and biomaterial properties. ECM, extracellular matrix.

Finally, clinicians should prioritize the selection of biomaterials based on their biological characteristics rather than their commercial availability. Collagen matrices currently represent the most evidence-based option for routine clinical use. In contrast, hydrogels, composite scaffolds, electrospun matrices, and biofunctionalized polymers should be considered emerging technologies with expanding but still evolving clinical indications [503,504] (Figure 5 and Table 12).

It should be emphasized that the algorithm presented here has been derived from the available literature and from clinical reasoning and has not been prospectively validated in a clinical study. It is therefore intended as a structured aid to decision-making rather than as a validated instrument, and prospective evaluation of its diagnostic and therapeutic performance remains necessary.

### 7.6. Future Clinical Perspective

It is anticipated that advancements in clinical decision-making will transcend the current practice of selecting individual biomaterials and evolve towards the development of patient-specific regenerative constructs. Advancements in polymer chemistry, artificial intelligence, digital treatment planning, additive manufacturing, and tissue engineering are anticipated to facilitate the fabrication of customized scaffolds, which are designed to align with the morphology of individual defects, the characteristics of soft tissue, the biological risk profile, and the regenerative capacity of the patient.

Rather than posing the question of whether a collagen matrix should replace a connective tissue graft, future clinicians may instead select personalized, multifunctional scaffolds combining structural polymers, extracellular matrix components, growth factors, extracellular vesicles, and immunomodulatory molecules within a single construct optimized for each patient [505,506,507].

## 8. Regulatory, Manufacturing, and Economic Barriers to Clinical Translation

The distance between a promising scaffold and a marketed product is governed as much by regulatory and manufacturing constraints as by biological performance, and these constraints explain much of the gap between the breadth of the preclinical literature and the narrowness of the clinically available range.

Under Regulation (EU) 2017/745, devices manufactured using non-viable tissues of animal origin or their derivatives fall under Annex VIII, Rule 18 and are generally classified as Class III, the highest risk class. For the porcine and bovine collagen matrices discussed in this review, this entails mandatory notified body involvement, full technical documentation assessment, demonstration of transmissible spongiform encephalopathy risk control, and continuous post-market clinical follow-up. The transition from the former Medical Device Directive has raised the evidentiary and financial burden substantially, and in dentistry it has been argued that the net effect will be a reduction in the number of available products and an increase in their prices, with resources diverted from product development toward regulatory affairs [451]. Devices that combine a scaffold with a growth factor, a drug or a cellular component face a further obstacle, since the ancillary medicinal or biological component may shift the product into a combination or advanced therapy pathway with correspondingly higher requirements.

Manufacturing imposes a second set of limits. Batch-to-batch variability is intrinsic to tissue-derived materials, and the terminal sterilization required for a marketed device can itself alter the properties on which biological performance depends, since irradiation and chemical sterilization modify collagen crosslinking and degradation kinetics. Scaffolds that depend on precise architecture, on defined ligand density, or on the retention of labile biological activity are correspondingly harder to produce reproducibly at scale than a simple porous matrix. Patient-specific constructs and bioprinted scaffolds raise the additional problem that a product manufactured to individual anatomy cannot be validated as a single batch, which is precisely the model that current device regulation is built around.

Economically, the relevant comparison is not the unit price of the substitute against zero but against the true cost of autogenous harvesting, which includes additional operating time, a second surgical site, analgesia, management of donor-site complications and, in some systems, the opportunity cost of longer chair time. A substitute that is more expensive per unit may still be economically rational where it shortens the procedure or avoids a palatal donor site and may not be where the clinical objective requires the volume stability that only autogenous tissue currently provides. Formal cost-effectiveness analyses in periodontal and peri-implant plastic surgery remain scarce, and this is a substantive gap in the evidence base rather than a peripheral one.

## 9. Future Perspectives for Soft Tissue Engineering in Periodontology

The scope of this review is restricted to periodontal and peri-implant soft tissue augmentation; mineralized tissue regeneration is therefore not reviewed in detail. Periodontal regeneration in the complete sense, however, requires the coordinated restoration of both compartments, and the mechanisms governing biomineralization, mineral nucleation and crystal growth, and the cellular control of matrix mineralization constitute a distinct field with its own materials, models and literature. Scaffolds designed to modulate these processes, including bifunctional and gradient constructs intended to address the soft and hard tissue interface simultaneously, represent an important direction for future work and are treated in dedicated reviews of that subject.

**Figure 6:**
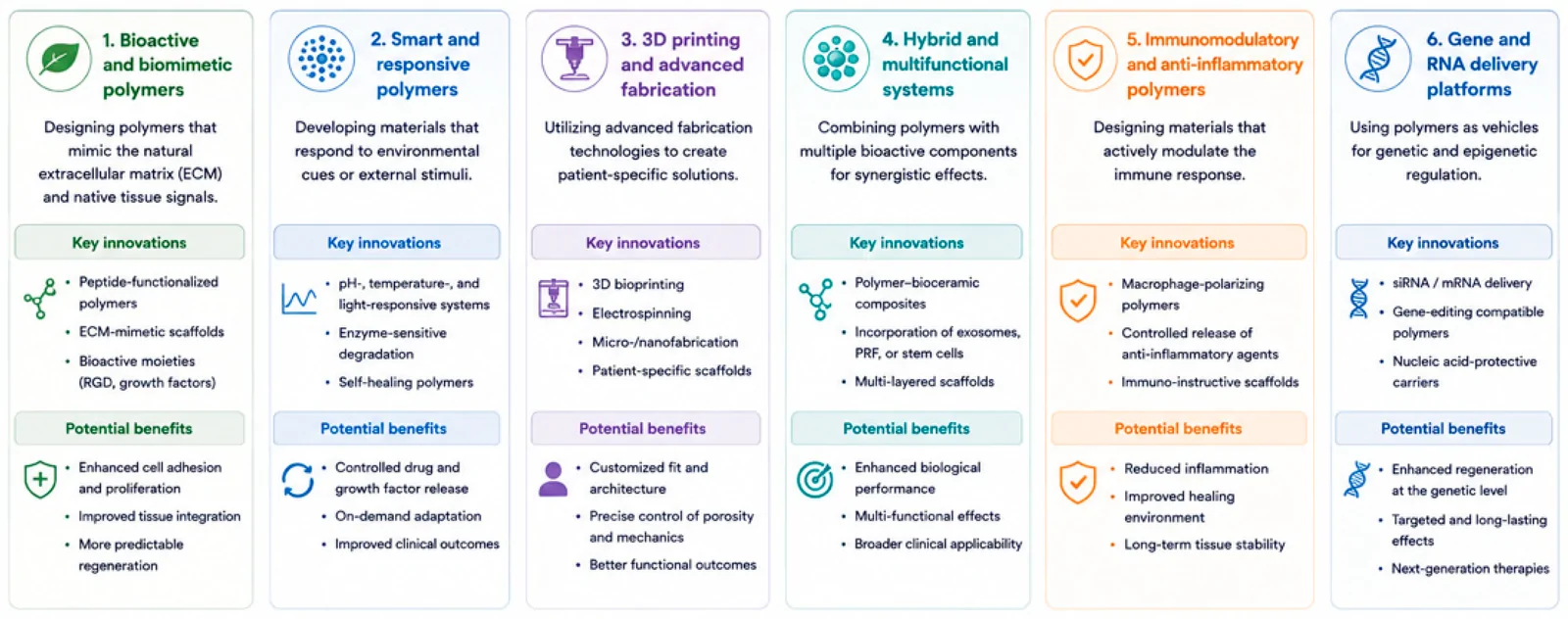
Future directions in polymer-based biomaterials for soft tissue regeneration, shown as six parallel developmental strands. For each strand, the figure gives the underlying concept, the key innovations, and the potential clinical benefits. (1) Bioactive and biomimetic polymers, designed to reproduce extracellular matrix architecture and native tissue signals through peptide functionalization and bioactive moieties such as RGD and growth factors. (2) Smart and responsive polymers, including pH-, temperature- and light-responsive systems, enzyme-sensitive degradation and self-healing materials. (3) Three-dimensional printing and advanced fabrication, encompassing bioprinting, electrospinning, micro- and nanofabrication, and patient-specific scaffolds. (4) Hybrid and multifunctional systems, combining polymers with bioceramics, exosomes, platelet-rich fibrin or stem cells in multi-layered constructs. (5) Immunomodulatory and anti-inflammatory polymers, which polarize macrophages and deliver anti-inflammatory agents from immuno-instructive scaffolds. (6) Gene and RNA delivery platforms, using polymers as carriers for siRNA and mRNA and as gene-editing-compatible, nucleic acid-protective vehicles. Each strand is color-coded consistently across its panels. RGD, arginine–glycine–aspartate; ECM, extracellular matrix; PRF, platelet-rich fibrin; siRNA, small interfering RNA; mRNA, messenger RNA.

Polymer-based biomaterials for periodontal and peri-implant soft tissue regeneration have evolved from passive volume replacement toward biologically active regenerative platforms. However, these materials still cannot fully replicate the biological complexity, mechanical performance, and long-term stability of autogenous connective tissue grafts. Consequently, future advancements are anticipated to emerge from the integration of polymer chemistry, tissue engineering, artificial intelligence, and precision medicine into multifunctional systems capable of actively directing regeneration. This shift is expected to transition the field from a focus on standardized biomaterial selection to a more personalized therapeutic approach [508] (Figure 6).

### 9.1. Smart Polymeric Biomaterials

Smart polymers, which possess the capacity to dynamically respond to the local biological environment, represent a particularly promising avenue of research in the field of regenerative biomaterials. In contrast to conventional static scaffolds, these materials exhibit the capacity to adapt their physical, chemical, or biological properties to internal or external stimuli. Adaptive polymers capable of self-healing, reversible crosslinking, or programmable degradation may further improve scaffold longevity and biological integration while reducing chronic foreign-body reactions [485].

A related development is four-dimensional printing, in which a printed construct is designed to change its shape or properties in a programmed manner after placement in response to physiological stimuli. Applied to adaptive tissue regeneration, this extends the stimulus-responsive principle from drug release to the geometry of the scaffold itself and may be relevant where a construct must accommodate the dimensional changes that follow soft tissue healing [509]. Comparable injectable and extrusion-printable hydrogel systems developed for controlled drug delivery in other soft tissue settings illustrate the formulation strategies that such constructs rely on [510].

### 9.2. Injectable Regenerative Scaffolds

Minimally invasive procedures are expected to assume an increasingly prominent role within the realm of periodontal and peri-implant therapy. Injectable polymeric scaffolds present a compelling alternative to surgically implanted materials, offering the potential to fill irregular defects while minimizing tissue trauma and operative complexity. The development and utilization of hydrogels for periodontal regeneration have undergone substantial advancement in recent years, particularly those derived from natural polymers such as alginate, collagen, and chitosan. Preclinical studies have demonstrated that the incorporation of these hydrogels with growth factors, stem cells, or other regenerative cues results in a consistent enhancement of cementum, periodontal ligament, and alveolar bone formation. This finding underscores their potential as pivotal components of next-generation periodontal therapies [486].

### 9.3. Personalized Biomaterials

The current state of biomaterials manufacturing involves the production of standardized products, which are intended for broad clinical application. However, an increasing recognition of patient-specific variability in tissue phenotype, inflammatory response, wound healing capacity, systemic health, and genetic background has stimulated growing interest in personalized regenerative medicine [489].

The employment of imaging technologies, such as CT scans, facilitates the generation of highly accurate 3D models of a patient’s bone structure. This, in turn, enables the fabrication of patient-specific polymer scaffolds that are customized to the defect’s precise shape and size [490,491].

This precision ensures that the scaffold fits perfectly into the defect site, promoting better mechanical stability and biological integration. The efficacy of the healing process is enhanced by optimizing the internal architecture and mechanical properties of these custom-made scaffolds, particularly in cases of complex fractures or substantial bone defects. This approach has been demonstrated to reduce recovery times and improve clinical outcomes, positioning personalized scaffolds as a critical innovation in advancing bone tissue engineering [492,493].

### 9.4. Artificial Intelligence in Biomaterial Design

The field of artificial intelligence (AI) is poised to bring about a fundamental transformation in the development, optimization, and clinical application of polymeric biomaterials. Machine learning algorithms have the capacity to analyze extensive datasets that describe polymer composition, molecular architecture, degradation behavior, mechanical properties, cellular responses, and clinical outcomes. Consequently, these algorithms can identify material combinations that may not be apparent through conventional experimental approaches [487].

The field of computational materials science has seen a notable increase in the use of artificial intelligence (AI) for predicting the performance of scaffolds prior to physical fabrication. This approach has been shown to reduce both the time required for development and the associated experimental costs. Algorithms have been developed to optimize polymer composition, crosslinking density, pore architecture, degradation profiles, and drug-release kinetics according to predefined biological objectives [488].

## 10. Conclusions and Outlook

The future of periodontal and peri-implant soft tissue regeneration will likely extend far beyond the concept of replacing autogenous grafts with synthetic substitutes. Instead, the development of intelligent biomaterials capable of actively orchestrating tissue regeneration is being driven by advances in polymer chemistry, tissue engineering, digital technologies, and computational sciences.

Smart polymers, injectable scaffolds, patient-specific biomaterials, and AI-assisted scaffold design represent complementary rather than competing technologies. The integration of these approaches holds the potential to transform regenerative dentistry from a procedure-based discipline into a biologically guided, precision-oriented therapeutic approach tailored to the individual characteristics of each patient.

Despite the persistent challenges in the scientific and regulatory domains, the rapid convergence of materials science, bioengineering, and clinical dentistry indicates that multifunctional polymeric scaffolds will become central components of future periodontal and peri-implant therapy. The translation of these innovations from experimental laboratories into predictable, evidence-based clinical practice will require continued interdisciplinary collaboration between clinicians, polymer chemists, materials scientists, and biomedical engineers.

## Figures and Tables

**Table 1:** Classification of polymer-based biomaterials. Polymers are grouped by main category (natural, synthetic, and hybrid/composite) and, additionally, according to structure and form. For each subcategory, representative examples, source or origin, key physicochemical and biological characteristics, typical fabrication forms, and representative clinical applications in soft tissue augmentation are summarized.

Main Category	Subcategory	Examples	Source/Origin	Key Characteristics	Typical Forms	Representative Clinical Applications	Evidence Stage	Ref.
Natural polymers	Collagen-based	Collagen, gelatin, elastin	Animal (bovine, porcine, marine)	Excellent biocompatibility Biodegradable Promotes cell adhesion Bioactive (cell-binding motifs)	Membranes, sponges, films, hydrogels, powders	Root coverage Keratinized tissue augmentation Peri-implant soft tissue augmentation Wound healing enhancement	Clinical—RCT (collagen); preclinical (gelatin, elastin)	[69,70,71,72,73,74,75,76,77,78,79,80,81,82,83,84,85,86,87]
	Polysaccharide-based	Hyaluronic acid (HA), chitosan, alginate, dextran	Natural (plants, algae, crustaceans, microbes)	Hydrophilic Biodegradable Tunable gelation Some have intrinsic bioactivity	Hydrogels, films, fibers, beads, microspheres	Soft tissue healing Phenotype modification Hydration and volume maintenance Drug delivery systems	Clinical—RCT (HA); preclinical (chitosan, alginate, dextran)	[88,89,90,91,92,93,94,95,96,97,98,99,100,101,102,103,104,105,106,107,108,109,110,111,112,113,114]
	Protein-based	Fibrin, silk fibroin	Human/animal (platelets, cocoon)	Excellent biocompatibility Bioactive Supports hemostasis Enzymatically degradable	Hydrogels, membranes, scaffolds	Early wound healing Soft tissue conditioning Adjunct in regenerative procedures	Clinical—RCT (fibrin, PRF); preclinical (silk fibroin)	[115,116,117,118,119,120,121,122,123,124]
Synthetic polymers	Aliphatic polyesters	PLA, PGA, PCL, PLGA	Petrochemical (synthetic)	Tunable degradation rate Good mechanical properties Processability FDA-approved polymers	Scaffolds, fibers, membranes, microspheres, 3D-printed structures	Guided tissue regeneration (GTR) Soft tissue scaffolds Volume maintenance Drug delivery	Preclinical; clinical use in dentistry largely confined to membranes and sutures	[125,126,127,128,129,130,131,132,133,134,135,136,137,138,139,140,141,142,143,144,145,146,147,148,149,150,151,152,153,154,155]
	Polyether	PEG, PEO, PPO	Petrochemical (synthetic)	Hydrophilic (PEG) Biocompatible Resistant to protein adsorption Chemically stable	Hydrogels, coatings, block copolymer systems	Hydrogel-based scaffolds Soft tissue augmentation Drug delivery Cell encapsulation	Preclinical; in vitro	[156,157,158,159,160,161]
	Other synthetic polymers	Polyurethane (PU), polycarbonates, polyanhydrides	Petrochemical (synthetic)	Versatile properties Tunable elasticity and strength Wide range of applications	Films, foams, elastomers, scaffolds, fibers	Soft tissue engineering; Elastic scaffolds; Complex tissue constructs	In vitro and preclinical	[162,163,164,165,166,167,168]
Hybrid/composite polymers	Natural–synthetic blends	Collagen/PLGA, gelatin/PCL, HA/PEG	Combination of natural and synthetic polymers	Balanced properties Improved mechanics Tunable degradation Enhanced bioactivity	Hydrogels, scaffolds, membranes, fibers	Soft tissue regeneration Contour augmentation Enhanced wound healing Drug/growth factor delivery	Preclinical	[169,170,171,172,173,174,175,176,177,178,179,180,181,182,183,184,185,186,187,188,189,190,191,192,193,194,195,196]
	Composite (polymers + inorganic)	Collagen/HA, PLGA/HA, chitosan/bioactive glass	Polymer + inorganic bioceramics	Increased mechanical strength Osteoconductive/bioactive Improved stability	Scaffolds, coatings, membranes	Soft tissue volume maintenance Peri-implant tissue integration Phenotype modification	Preclinical	[169,170,171,172,173,174,175,176,177,178,179,180,181,182,183,184,185,186,187,188,189,190,191,192,193,194,195,196]
	Functionalized polymers	Methacrylated collagen (GelMA), PEG–peptide, RGD-modified polymers	Chemically modified polymers	Enhanced cell interaction Targeted bioactivity Tunable surface properties	Hydrogels, scaffolds, coatings	Advanced tissue engineering Cell adhesion and proliferation Bioactive scaffolds	In vitro and preclinical	[169,170,171,172,173,174,175,176,177,178,179,180,181,182,183,184,185,186,187,188,189,190,191,192,193,194,195,196]
According to structure and form	Hydrogels	HA hydrogels, GelMA, PEG hydrogels	Natural, synthetic or hybrid	High water content Excellent biocompatibility Injectable (in some cases)	Injectable gels, thermo/photocurable gels	Minimally invasive augmentation Soft tissue regeneration Drug/growth factor delivery	Clinical—RCT (HA hydrogels); preclinical (GelMA, PEG hydrogels)	[88,89,90,91,92,93,94,95,96,97,98,151,152,153,154,155,156,157,158,159,160,161]
	Fibrous scaffolds	Electrospun collagen, PCL, PLGA	Natural, synthetic or hybrid	Mimics ECM structure; High surface area; Supports cell alignment	Non-woven mats, electrospun fibers	Soft tissue engineering; Guided tissue regeneration; Wound healing	Preclinical	[197,198,199,200,201,202,203,204,205,206]
	Porous scaffolds	PLGA, PCL, collagen sponges	Natural, synthetic or hybrid	High porosity; Cell infiltration; Vascularization potential	Sponges, foams, 3D-printed scaffolds	Volume augmentation Soft tissue reconstruction Peri-implant augmentation	Clinical—RCT (collagen sponges); preclinical (PLGA, PCL)	[69,70,71,72,73,74,75,76,77,78,79,150,151,152,153,154,155]
	Membranes/films	Collagen membranes, PLA films	Natural, synthetic or hybrid	Barrier function; Easy handling; Space maintenance	Resorbable membranes, barrier films	Barrier in regenerative procedures Space maintenance Wound protection	Clinical—RCT (collagen membranes); preclinical (PLA films)	[69,70,71,72,73,74,75,76,77,78,79,125,126,127,128,129,130,131,132,133,134]
	Particles/microspheres	PLGA microspheres, chitosan nanoparticles	Natural, synthetic or hybrid	Controlled release Protects bioactive molecules Injectable formulations	Microspheres, nanoparticles	Controlled drug delivery Anti-inflammatory/antimicrobial Bioactive molecule delivery	Preclinical; in vitro	[99,100,101,102,103,104,105,106,150,151,152,153,154,155]

**Evidence stage.** The evidence stage column reports the highest stage of investigation reached for the materials in that row in periodontal or peri-implant soft tissue applications, using the categories in vitro, preclinical (animal model), clinical–case series, and clinical–randomized controlled trial. Where materials within a row differ, the stage is given per material. This column is provided so that the representative applications listed are not read as evidence of established clinical use for every material in the row. ECM—extracellular matrix; GelMA—gelatin methacryloyl; GTR—guided tissue regeneration; HA—hyaluronic acid; PCL—polycaprolactone; PEG—polyethylene glycol; PEO—polyethylene oxide; PGA—polyglycolic acid; PLA—polylactic acid; PLGA—poly(lactic-co-glycolic acid); PPO—polypropylene oxide; PU—polyurethane; RGD—arginine–glycine–aspartic acid.

**Table 2:** Natural polymers. Origin, principal advantages, limitations, and current clinical use of the naturally derived polymers most frequently applied in periodontal and peri-implant soft tissue regeneration: collagen, hyaluronic acid, gelatin, fibrin, extracellular matrix (ECM) derivatives, chitosan, and alginate.

Polymer	Origin	Advantages	Limitations	Clinical Use	Evidence Stage	Ref.
Collagen	Animal connective tissues (bovine, porcine, equine, marine)	Excellent biocompatibility and low immunogenicity Promote cell adhesion, migration and proliferation Biodegradable and resorbable Supports angiogenesis and ECM remodeling Extensive clinical experience	Moderate mechanical stability Enzymatic degradation Batch-to-batch variability Risk of disease transmission (animal origin)	Root coverage procedures Soft tissue augmentation (KTW) Periodontal phenotype modification Peri-implant soft tissue augmentation Wound healing enhancement	Clinical—RCT and meta-analysis	[69,70,71,72,73,74,75,76,77,78,79]
Hyaluronic acid (HA)	Widely distributed in connective tissues, synovial fluid, and ECM	Highly hydrophilic maintains tissue hydration Modulates inflammation and immune response Promotes cell migration and proliferation Angiogenic and anti-inflammatory properties Available in different molecular weights	Rapid in vivo degradation (short residence time) Low mechanical strength Requires crosslinking for prolonged stability Effect depends on molecular weight	Adjunct in root coverage procedures Soft tissue wound healing Management of gingival recession Peri-implant soft tissue healing Hydration and volume maintenance	Clinical—RCT	[88,89,90,91,92,93,94,95,96,97,98]
Gelatin	Denatured collagen (derived from bovine or porcine collagen)	Excellent biocompatibility and biodegradability RGD sequences promote cell attachment Easy to process into hydrogels, films, sponges Good drug and growth factor carrier Low cost and high availability	Lower mechanical strength Faster degradation Thermosensitive (gel–sol transition) Potential immunogenicity at high doses	Injectable hydrogels for soft tissue defects Drug/growth factor delivery systems Wound healing enhancement Adjunct in regenerative procedures Experimental tissue engineering	Preclinical; in vitro	[80,81,82,83,84,85,86,87]
Fibrin	Human blood (plasma fibrinogen converted to fibrin)	Natural provisional matrix of wound healing Promotes cell migration and angiogenesis Contains endogenous growth factors (when autologous) Excellent biocompatibility Easily prepared from autologous blood	Rapid degradation Low mechanical stability Limited volume maintenance Donor variability (autologous preparations)	Adjunct in soft tissue surgery Combination with grafts or membranes Wound healing acceleration Socket/wound protection Peri-implant soft tissue healing	Clinical—RCT	[115,116,117,118,119,120,121,122,123,124]
Extracellular matrix (ECM) derivatives	Decellularized human or animal tissues (dermis, urinary bladder matrix, pericardium, serosa, etc.)	Preserves native ECM architecture and bioactive cues Promotes cell infiltration and angiogenesis Low immunogenicity due to decellularization Supports tissue remodeling and integration Good biological signaling capacity	Variable composition and source Complex and costly manufacturing Limited long-term clinical data Regulatory and standardization challenges	Soft tissue augmentation Peri-implant soft tissue reconstruction Mucosal defect coverage Ridge contour enhancement Emerging applications	Clinical—RCT	[214,215,216,217,218,219,220,221,222]
Chitosan	Deacetylated chitin (from crustacean shells or fungal cell walls)	Biocompatible and biodegradable Antimicrobial, antifungal and hemostatic properties Promotes cell adhesion and proliferation Easily modified (chemical functionality) Good film-forming and gel-forming ability	Poor solubility at neutral pH Batch variability Lower mechanical strength Possible allergic reactions (shellfish origin)	Wound dressings and membranes Soft tissue regeneration adjunct Drug and antimicrobial delivery Guided tissue regeneration (adjunct) Experimental scaffolds and hydrogels	Preclinical; limited clinical case series	[99,100,101,102,103,104,105,106]
Alginate	Brown seaweed (alginic acid salts)	Biocompatible and minimally immunogenic Mild gelation under physiological conditions High water content maintains moist environment Easily formed into hydrogels and beads Good for cell encapsulation	Lacks cell-adhesion motifs (requires modification) Lower mechanical strength Rapid ion exchange and disintegration Limited long-term stability in vivo	Injectable hydrogels for soft tissue engineering Cell and growth factor delivery Wound filling and protection Experimental 3D bioprinting bioinks Adjunct in regenerative therapy	In vitro and preclinical	[107,108,109,110,111,112,113,114]

**Evidence stage.** The evidence stage column reports the highest stage of investigation reached for the materials in that row in periodontal or peri-implant soft tissue applications, using the categories in vitro, preclinical (animal model), clinical–case series, and clinical–randomized controlled trial. Where materials within a row differ, the stage is given per material. This column is provided so that the representative applications listed are not read as evidence of established clinical use for every material in the row. ECM—extracellular matrix; KTW—keratinized tissue width; RGD—arginine–glycine–aspartic acid peptide sequence.

**Table 3:** Synthetic polymers. Chemical structure, origin or constituent monomer, advantages, limitations, and current clinical status of the synthetic polymers investigated for soft tissue regeneration: polylactic acid (PLA), polyglycolic acid (PGA), polycaprolactone (PCL), polyethylene glycol (PEG), poly(lactic-co-glycolic acid) (PLGA), and polyurethane (PU).

Polymer	Repeating Unit	Origin/Monomer	Advantages	Limitations	Clinical Use (Current Status)	Ref.
Polylactic acid (PLA)	[–O–CH(CH_3_)–CO–]_n_	Lactic acid (from renewable resources: corn, sugarcane, etc.)	Excellent biocompatibility Good mechanical strength and stiffness Predictable degradation rate FDA-approved Easy to process (electrospinning, 3D printing, etc.)	Relatively hydrophobic Slow degradation (months–years) Brittleness and low elongation Acidic degradation products (lactic acid) may cause local pH decrease	Research in soft tissue engineering scaffolds Drug delivery systems Not yet routinely used in periodontal soft tissue augmentation	[125,126,127,128,129,130,131,132,133,134]
Polyglycolic acid (PGA)	[–O–CH_2_–CO–]_n_	Glycolic acid (synthetic)	High hydrophilicity Good biocompatibility Fast degradation High tensile strength Well established in clinical sutures	Very rapid degradation (weeks–months) Poor mechanical stability Brittle Acidic by-products (glycolic acid) may cause inflammation	Used in absorbable sutures (very limited use in soft tissue regeneration) Research in fast-degrading scaffolds and drug delivery	[135,136,137,138,139,140,141]
Polycaprolactone (PCL)	[–O–(CH_2_)_5_–CO–]_n_	ε-Caprolactone (synthetic)	Excellent flexibility and toughness Very slow degradation (years) Hydrophobic—good for long-term structural support Easy to process Low melting point	Very hydrophobic (poor cell adhesion) Very slow degradation Low protein adsorption Not bioactive	Research in long-term scaffolds for soft tissue regeneration 3D-printed scaffolds Drug delivery systems Not yet clinically applied in periodontal procedures	[142,143,144,145,146,147,148,149]
Polyethylene glycol (PEG)	[–CH_2_–CH_2_–O–]_n_	Ethylene oxide (synthetic)	Highly hydrophilic Excellent biocompatibility Low immunogenicity Chemically versatile (easy functionalization) Used in hydrogels	Non-biodegradable (unless modified) Bioinert (lacks cell-adhesion sites) Poor mechanical strength in pure form	Injectable hydrogels Cell encapsulation Drug and growth factor delivery Experimental soft tissue engineering	[156,157,158,159,160,161]
Poly(lactic-co-glycolic acid) (PLGA)	[–O–CH(CH_3_)–CO–]_x_[–O–CH_2_–CO–]_γ_	Lactic acid + glycolic acid (copolymer)	Tunable degradation rate by lactic/glycolic ratio FDA-approved Good mechanical properties Widely used in drug delivery Versatile fabrication	Acidic degradation products Possible initial burst release of encapsulated drugs Mechanically weaker than some other polyesters	Drug delivery (growth factors, antibiotics, etc.) Research in soft tissue engineering scaffolds Experimental use in periodontal regeneration	[150,151,152,153,154,155]
Polyurethane (PU)	[–NH–CO–O–R–]_n_ (carbamate linkage)	Diisocyanates + polyols (synthetic)	Excellent elasticity and toughness High wear and fatigue resistance Tunable mechanical properties Versatile chemistry Can be biodegradable (if designed)	Complex synthesis Potential cytotoxicity (residual monomers) Degradation by-products depend on formulation	Research in elastic scaffolds and films for soft tissue engineering Wound dressings Very limited clinical application in periodontology (experimental)	[162,163,164,165,166,167,168]

Repeating units are given in simplified linear notation; n, x, and y denote the number of repeating units. FDA—United States Food and Drug Administration.

**Table 4:** Mechanical properties of polymer-based biomaterials used in soft tissue augmentation. Tensile strength, elastic modulus, elongation at break, approximate degradation rate, typical mechanical behavior, and the resulting clinical implications are compared across natural, synthetic, and hybrid/composite polymers. Degradation rate is approximate and depends on molecular weight, crosslinking, crystallinity, scaffold architecture, and the in vivo environment.

Polymer Type	Example Material	Tensile Strength (MPa)	Elastic Modulus (MPa)	Elongation at Break (%)	Degradation Rate *	Typical Mechanical Behavior	Clinical Implications	Ref.
Natural	Collagen	0.5–5	0.1–10	10–60	Days–weeks	Soft, flexible, low strength	Excellent biocompatibility but requires reinforcement for volume stability	[69,70,71,72,73,74,75,76,77,78,79]
	Hyaluronic acid (HA)	0.02–0.2	0.01–0.5	50–300	Days–weeks	Viscoelastic, highly hydrophilic	Ideal for hydration and early wound healing; limited mechanical support	[88,89,90,91,92,93,94,95,96,97,98]
	Gelatin	0.1–2	0.05–2	20–150	Days–weeks	Soft, elastic, thermosensitive	Good for cell attachment and drug delivery; rapid resorption	[80,81,82,83,84,85,86,87]
	Fibrin	0.01–0.5	0.001–0.2	5–25	Days	Very soft, brittle	Provides hemostasis and biological activity; minimal structural support	[115,116,117,118,119,120,121,122,123,124]
	Chitosan	5–100	10–500	10–40	Weeks–months	Stiff, strong, moderately brittle	Good mechanical support; antimicrobial; limited elasticity	[99,100,101,102,103,104,105,106]
	Alginate	0.05–1	0.01–0.2	20–80	Days–weeks	Soft, gel-like	Good for hydrogels and cell encapsulation; low strength	[107,108,109,110,111,112,113,114]
Synthetic	PLA	50–70	1000–3500	2–6	Months–years	Stiff, strong, brittle	Provides structural support; slow degradation	[125,126,127,128,129,130,131,132,133,134]
	PGA	30–60	1000–3000	3–10	Weeks–months	Stiff, brittle	Fast-degrading; suitable for temporary support	[135,136,137,138,139,140,141]
	PLGA (50:50)	20–60	500–2500	5–20	Weeks–months	Moderately stiff, less brittle	Widely used; tunable degradation and mechanical properties	[150,151,152,153,154,155]
	PCL	10–25	200–500	100–800	Years	Flexible, tough, elastic	Excellent elasticity and long-term structural integrity	[142,143,144,145,146,147,148,149]
	PEG (hydrogels)	0.01–0.5	0.01–1	100–1000	Stable (non-degradable) or weeks–months (if crosslinked)	Soft, viscoelastic	Ideal for injectable systems and cell delivery	[156,157,158,159,160,161]
	PU	5–50	1–100	100–1000	Months–years (tunable)	Elastic, tough, versatile	Properties highly tunable for specific applications	[162,163,164,165,166,167,168]
Hybrid/composite	Collagen/PLGA	5–30	50–500	20–100	Weeks–months	Balanced stiffness and elasticity	Improved strength and stability with maintained biocompatibility	[169,170,171,172,173,174,175,176,177,178,179,180,181,182,183,184,185,186,187,188,189,190,191,192,193,194,195,196]
	Gelatin/PCL	5–20	20–200	50–300	Months–years	Flexible, resilient	Suitable for long-term soft tissue regeneration	[169,170,171,172,173,174,175,176,177,178,179,180,181,182,183,184,185,186,187,188,189,190,191,192,193,194,195,196]
	HA/PEG	0.02–1	0.01–5	100–500	Weeks–months	Soft, hydrated, viscoelastic	Excellent for injectable hydrogels and volume maintenance	[169,170,171,172,173,174,175,176,177,178,179,180,181,182,183,184,185,186,187,188,189,190,191,192,193,194,195,196]
	Functionalized polymers	Variable (5–50)	Variable (10–1000)	Variable (10–300)	Tunable	Tunable (from soft to stiff)	Properties tailored by chemical modification and crosslinking	[169,170,171,172,173,174,175,176,177,178,179,180,181,182,183,184,185,186,187,188,189,190,191,192,193,194,195,196]

* Degradation rate is approximate and depends on molecular weight, crosslinking, crystallinity, scaffold architecture, and the in vivo environment. MPa—megapascals; PLA—polylactic acid; PGA—polyglycolic acid; PLGA—poly(lactic-co-glycolic acid); PCL—polycaprolactone; PEG—polyethylene glycol; PU—polyurethane; HA—hyaluronic acid.

**Table 5:** Biological effects of polymer-based biomaterials relevant to soft tissue regeneration. Reported effects on angiogenesis (endothelial cell proliferation, new vessel formation, pro-angiogenic factor release), fibroblast behavior (adhesion and spreading, proliferation, collagen synthesis), and immune response (macrophage modulation, inflammation level, overall immune effect) are summarized for each polymer class. Arrows denote the direction and relative magnitude of the effect, as defined in the table footnote.

Polymer Type	Example Material	Endothelial Cell Proliferation	New Vessel Formation	Pro-Angiogenic Factor Release	Fibroblast Adhesion and Spreading	Fibroblast Proliferation	Collagen Synthesis	Macrophage Modulation	Inflammation Level	Overall Immune Effect	Ref.
Natural	Collagen	↑↑	↑↑	↑↑	↑↑	↑↑	↑↑	M2 ↑↑	↓↓	Pro-regenerative (immunotolerant)	[69,70,71,72,73,74,75,76,77,78,79]
	Hyaluronic acid (HA)	↑↑	↑↑	↑	↑	↑	↑	M2 ↑	↓	Anti-inflammatory, pro-healing	[88,89,90,91,92,93,94,95,96,97,98]
	Gelatin	↑	↑	↑	↑↑	↑	↑	M2 ↑	↓	Favors tissue repair	[80,81,82,83,84,85,86,87]
	Fibrin	↑↑	↑↑	↑↑	↑↑	↑↑	↑	M2 ↑	↓↓	Immunomodulatory, pro-hemostatic	[115,116,117,118,119,120,121,122,123,124]
	Chitosan	↑	↑	↑	↑	↑	↑	M1/M2 balance (M2 ↑)	↓	Mildly anti-inflammatory, antimicrobial	[99,100,101,102,103,104,105,106]
	Alginate	↑	↑	–	↑	↑	–	M2 ↑	↓	Well tolerated	[107,108,109,110,111,112,113,114]
Synthetic	PLA	↑	↑	–	↑	↑	↑	M1/M2 balance	=/↓	Generally neutral	[125,126,127,128,129,130,131,132,133,134]
	PGA	↑	↑	–	↑	↑	–	M1/M2 balance	=/↓	Generally neutral	[135,136,137,138,139,140,141]
	PLGA	↑	↑	–	↑	↑	↑	M2 ↑ (with degradation)	↓	Biocompatible	[150,151,152,153,154,155]
	PCL	–	–	–	↑	–	–	M1/M2 balance	=	Biologically inert	[142,143,144,145,146,147,148,149]
	PEG (hydrogels)	↑ (if modified)	↑ (if modified)	↑ (if modified)	↑ (if RGD)	↑ (if modified)	↑ (if modified)	M2 ↑ (if modified)	↓ (if modified)	Tunable	[156,157,158,159,160,161]
	PU	↑	↑	–	↑	↑	↑	M1/M2 balance	=/↓	Depends on chemistry	[162,163,164,165,166,167,168]
Hybrid/composite	Collagen/PLGA	↑↑	↑↑	↑	↑↑	↑↑	↑↑	M2 ↑	↓	Enhanced regeneration	[169,170,171,172,173,174,175,176,177,178,179,180,181,182,183,184,185,186,187,188,189,190,191,192,193,194,195,196]
	Gelatin/PCL	↑	↑	↑↑	↑↑	↑	↑	M2 ↑	↓	Favorable	[169,170,171,172,173,174,175,176,177,178,179,180,181,182,183,184,185,186,187,188,189,190,191,192,193,194,195,196]
	HA/PEG	↑↑	↑↑	↑↑	↑↑	↑↑	↑	M2 ↑↑	↓↓	Strongly pro-regenerative	[169,170,171,172,173,174,175,176,177,178,179,180,181,182,183,184,185,186,187,188,189,190,191,192,193,194,195,196]
	Functionalized polymers	↑↑ (tunable)	↑↑ (tunable)	↑↑ (tunable)	↑↑ (tunable)	↑↑ (tunable)	↑↑ (tunable)	M2 ↑↑ (tunable)	↓↓ (tunable)	Highly tunable (immunomodulatory)	[169,170,171,172,173,174,175,176,177,178,179,180,181,182,183,184,185,186,187,188,189,190,191,192,193,194,195,196]

**System tested.** Unless otherwise indicated, entries refer to the polymer in the unmodified form in which it is used for scaffold fabrication. Entries for PEG refer to functionalized PEG hydrogels, and the qualifiers “if modified” and “if RGD” denote effects reported only for peptide-conjugated or otherwise chemically modified formulations; unmodified PEG is protein-resistant and does not support these responses. Entries for functionalized polymers refer to systems conjugated with peptides, growth factors or antimicrobial moieties, and the qualifier “tunable” indicates that the magnitude of the effect depends on the ligand and its density. The hybrid and composite rows refer to the specific combinations named in the Example material column. **References.** References are given at the level of the section in which the biological effects of each material are discussed in detail, since most entries are supported by several studies rather than by a single source. Effects reported for the hybrid and composite systems are discussed together in Section 5.1. ↑↑ strong increase; ↑ moderate increase; – minimal or no effect; ↓↓ strong decrease; ↓ moderate decrease; = no significant change. M1—pro-inflammatory macrophage phenotype; M2—anti-inflammatory/pro-regenerative macrophage phenotype. Functionalization examples: RGD peptides, growth factors, ionic groups, antioxidant or anti-inflammatory moieties.

**Table 6:** Biological–polymer–functional agent interactions in soft tissue regeneration. For each functional domain, the table gives the polymer or agent presenting it, the cellular or molecular partner engaged, the biological function tailored, and the biological requirement (Section 2) to which it corresponds. Motifs marked as introduced by biofunctionalization are not intrinsic to the base polymer and must be conjugated during scaffold fabrication.

Functional Domain/Motif	Polymer or Agent Presenting It	Cellular or Molecular Partner	Biological Function Tailored	Corresponding Requirement (Section)	Ref.
RGD (Arg-Gly-Asp)	Fibrin, gelatin; introduced into PEG, PCL, PLGA by biofunctionalization	Integrins (αvβ3, α5β1)	Fibroblast and keratinocyte adhesion, spreading and migration; ligand density and spacing govern focal adhesion formation	Fibroblast migration and ECM formation (2.4); epithelial integration (2.5)	[287]
GFOGER and related GxOGER motifs	Native fibrillar collagen; collagen matrices	Collagen-binding integrins α1β1, α2β1	High-affinity adhesion in triple-helical conformation; lost if the helix is denatured	Fibroblast migration and ECM formation (2.4)	[288]
Glycosaminoglycan backbone (HA)	Hyaluronic acid; HA-containing composites	CD44, RHAMM	Molecular-weight-dependent modulation of inflammation and cell migration; hydration of the wound bed	Immunomodulation and macrophage polarization (2.2); angiogenesis (2.3)	[88,89,90,91,92,93,94,95,96,97,98,289]
Fibrin αC and RGD domains, platelet factors	Fibrin, PRF, autologous platelet concentrates	Platelet and leukocyte integrins; endogenous growth factors	Haemostasis, provisional matrix formation, early angiogenic signaling	Haemostasis and early inflammatory response (2.1); angiogenesis (2.3)	[115,116,117,118,119,120,121,122,123,124]
Cationic amino groups (degree of deacetylation)	Chitosan and chitosan composites	Anionic bacterial membranes; macrophage surface receptors	Antimicrobial and hemostatic activity; shift in macrophage phenotype towards M2	Haemostasis (2.1); immunomodulation (2.2)	[99,100,101,102,103,104,105,106]
Sulfated/heparin-mimetic domains	Heparin, sulfated GAGs, starPEG–heparin hydrogels	Heparin-binding domains of VEGF, FGF-2, SDF-1α	Affinity-based sequestration, protection and demand-driven release of growth factors	Angiogenesis (2.3); controlled biodegradation (2.7)	[290]
MMP-cleavable peptide crosslinks	PEG and hybrid hydrogels (biofunctionalization)	Matrix metalloproteinases secreted by infiltrating cells	Cell-mediated degradation synchronized with tissue ingrowth rather than fixed hydrolysis	Controlled biodegradation (2.7); mechanical stability (2.6)	[156,157,158,159,160,161,169,170,171,172,173,174,175,176,177,178,179,180,181,182,183,184,185,186,187,188,189,190,191,192,193,194,195,196]
Protein-resistant ether backbone	PEG, PEO	None (suppresses non-specific protein adsorption)	Blank slate: minimal fouling and low intrinsic bioactivity, permitting fully defined ligand presentation	Design of an ideal polymeric scaffold (2.8)	[156,157,158,159,160,161]

**Table 7:** Clinical indications for polymer-based biomaterials in periodontal and peri-implant soft tissue augmentation. Two distinct scales are reported: the strength of the available clinical evidence, and the anticipated clinical predictability for each of seven indications. Grading criteria for both scales are defined beneath the table.

Polymer Category	Example	Evidence Level	Root Coverage	Phenotype Modification	Keratinized Tissue Augmentation	Peri-Implant Soft Tissue Augmentation	Ridge Contour/Volume Maintenance	Wound Healing Adjunct	Socket/Soft Tissue Defect Coverage	Remarks
Natural	Collagen (matrices, membranes)	High [404,405,406,407]	High	High	Moderate	High	Moderate	High	High	Most clinically established alternative to CTG in selected cases
	Hyaluronic acid (HA)	Moderate [177,185]	Moderate	Moderate	Low–moderate	Moderate	Low	High	Moderate	Excellent adjunct for healing and hydration
	Gelatin	Low	Moderate	Moderate	Low–moderate	Moderate	Low–moderate	High	Moderate	Good cell-interactive properties; faster resorption
	Fibrin/PRF	Moderate [195,198]	Low–moderate	Low–moderate	Low	Low–moderate	Low	High	High	Biological glue and healing enhancer; not a volume replacement
	Chitosan	Low [216]	Low–moderate	Low–moderate	Low–moderate	Low–moderate	Moderate	Moderate	Moderate	Antimicrobial and hemostatic potential
	Alginate	Low	Low	Low	Low	Low	Low–moderate	Low–moderate	Low–moderate	Hydrogels useful as injectable carriers
Synthetic	PLA	Low	Low	Low	Low	Low–moderate	High	Low–moderate	Low–moderate	Mainly for structural support; long resorption time
	PGA	Low	Low	Low	Low	Low	Moderate	Low	Low	Fast-degrading; limited clinical use
	PLGA	Low	Low	Low	Low	Low–moderate	Moderate	Low	Low	Tunable degradation and mechanics
	PCL	Low [257]	Low	Low	Low	Low–moderate	High	Low	Low	Excellent long-term mechanical stability
	PEG (hydrogels)	Low	Low–moderate	Moderate	Low–moderate	Moderate	Low–moderate	High	Moderate	Injectable systems for minimally invasive procedures
	PU	Low	Low	Low	Low	Low	Low–moderate	Low	Low	Research stage; promising elasticity
Hybrid/composite	Collagen/PLGA	Low	Moderate	Moderate	Moderate	High	High	High	High	Balanced bioactivity and mechanical properties
	Gelatin/PCL	Low	Moderate	Moderate	Moderate	High	High	Moderate–high	High	Promising for long-term soft tissue regeneration
	HA/PEG	Low	Moderate	High	Moderate	Moderate–high	Moderate	High	Moderate	Injectable, bioactive and hydrating
	Functionalized polymers	Low	Moderate–high	High	Moderate–high	High	High	High	High	Tailored bioactivity (growth factors, antimicrobial, etc.)

**Evidence level.** Grades the strength of the clinical evidence available for the material specifically in a periodontal or peri-implant soft tissue augmentation indication. High, at least one systematic review or meta-analysis of randomized controlled trials in such an indication; Moderate, at least one randomized controlled trial in such an indication, or a systematic review of non-randomized or adjunctive studies; Low, case series, preclinical or in vitro evidence only. Evidence relating to other applications, including guided bone regeneration and general dental use, was not counted towards this grade. Key supporting references are given in the column where a specific source could be identified; where no reference is given, the grade denotes the absence of clinical trial evidence in these indications, and the supporting materials-science literature is cited in the relevant sections. **Anticipated clinical predictability.** The seven indication columns grade the expected performance of the material for each indication, based on the material properties and biological rationale set out in Section 2, Section 3, Section 4 and Section 5 together with the available evidence. These grades represent the consensus judgement of the authors and are not derived from a formal appraisal instrument. They are reported separately from the evidence level because a material may have a strong biological rationale for an indication while the clinical evidence supporting its use in that indication remains limited, and the converse also occurs. **Abbreviations and notes.** Functionalized polymers are polymers modified with growth factors, peptides, antimicrobial agents or other bioactive moieties. CTG—connective tissue graft; HA—hyaluronic acid; PRF—platelet-rich fibrin; PLA—polylactic acid; PGA—polyglycolic acid; PLGA—poly(lactic-co-glycolic acid); PCL—polycaprolactone; PEG—polyethylene glycol; PU—polyurethane.

**Table 8:** Advantages of polymer-based biomaterials over connective tissue grafts (CTG). Each advantage is described and compared directly between polymer-based biomaterials and CTG, with the corresponding clinical impact or benefit indicated.

Advantage	Description	Polymer-Based Biomaterials	CTG	Clinical Impact/Benefit	Evidence Support	Ref.
No donor site surgery	Eliminates the need for harvesting tissue from the palate or other intraoral sites.	No additional surgical site Reduced operative time No donor site morbidity	Requires donor site surgery Additional surgical trauma More complex procedure	Less invasive treatment Higher patient acceptance Simplified surgical protocol	High	[409,418,419,450]
Reduced patient morbidity	Avoids postoperative pain, bleeding, discomfort, and complications associated with the donor site.	Less postoperative pain; Minimal bleeding; Lower risk of complications	Higher postoperative pain Palatal bleeding and discomfort Possible complications (ulceration, infection, paresthesia)	Improved patient comfort; Faster recovery; Better overall experience	High	[418,419,450]
Shorter operative time	Simplifies the surgical procedure and reduces chair time.	No harvesting time; Fewer surgical steps; Generally faster procedure	Additional time required for harvesting; More complex technique	Increased clinical efficiency Lower cost per procedure Improved practice workflow	Moderate	[450]
No donor site complications	Eliminates risks related to the harvesting site.	No risk of palatal ulceration No risk of infection No risk of neurosensory alterations	Risk of palatal ulceration Risk of infection Risk of neurosensory disturbances	Enhanced patient safety Predictable postoperative course Fewer emergency visits	Moderate	[418,419,450]
Faster soft tissue healing	Many polymeric biomaterials promote early wound stabilization and epithelial coverage.	Supports early wound healing Promotes re-epithelialization Less inflammation	Slower epithelialization at donor site Greater postoperative inflammation	Accelerated soft tissue recovery Earlier return to normal function Better esthetic outcomes	Low	[408,409,410,411,412,413,414,415,416,417]
Minimally invasive and adaptable delivery	Available in various forms (membranes, hydrogels, injectable systems) that adapt to the defect.	Multiple delivery formats Conforms to complex defects Injectable options available	Requires flap elevation and precise suturing Limited adaptability of graft	Better adaptation to defect morphology Improved handling in challenging sites Possibility of minimally invasive approaches	Low	[437,438,439,440,441,442,443,444,445,446]
Potential for biological bioactivity	Can be engineered to deliver growth factors, peptides, or other bioactive molecules.	Can be biofunctionalized Supports cell migration Can deliver therapeutic agents	No intrinsic bioactivity beyond autologous tissue No controlled release capability	Enhanced regenerative potential Customizable biological response Next-generation therapeutics	Low	[279,280,281,282,283,284,285,286]
Off-the-shelf availability and standardization	Commercially available with consistent quality and characteristics.	Ready to use; Standardized quality; No variability in donor tissue	Patient-to-patient variability Depends on tissue availability Technique-sensitive	Predictable material properties Easier inventory management Reproducible outcomes	n/a	[406,407,447,448,449]
Better acceptance in certain patient groups	Particularly beneficial for patients refusing donor site surgery or with limited tissue volume.	Preferred by many patients Suitable for medically compromised patients Option in limited donor tissue	Some patients refuse donor site Not ideal in limited palatal tissue Higher surgical burden	Increased treatment acceptance Broader indication range Personalized treatment options	Moderate	[418,419,450]

**Evidence support.** Grades the strength of the clinical evidence supporting the stated comparison, using the definitions given beneath Table 7: High, at least one systematic review or meta-analysis of randomized controlled trials in a periodontal or peri-implant soft tissue augmentation indication; Moderate, at least one randomized controlled trial in such an indication, or a systematic review of non-randomized or adjunctive studies; Low, case series, preclinical or in vitro evidence only. Entries marked n/a denote practical, economic or regulatory considerations rather than clinical outcomes, for which the concept of a level of clinical evidence does not apply. These categories are descriptive evidence tiers based on study design and availability; they do not represent a formal certainty-of-evidence assessment (for example, GRADE) or a formal risk-of-bias appraisal. CTG—connective tissue graft.

**Table 9:** Disadvantages and current limitations of polymer-based biomaterials compared with connective tissue grafts (CTG). Each limitation is described and compared directly between polymer-based biomaterials and CTG, with the corresponding clinical impact indicated.

Disadvantage/Limitation	Description	Polymer-Based Biomaterials	CTG	Clinical Impact	Evidence Support	Ref.
Limited long-term predictability	Long-term stability and durability are still inferior to autogenous grafts in many cases.	Greater volume reduction over time Less stable root coverage Limited long-term clinical data	Excellent long-term stability Predictable volume maintenance Extensive long-term evidence	Possible need for retreatment Uncertain outcomes in severe defects Caution in high esthetic areas	High	[409,450]
Lower volumetric stability	Higher resorption rate may lead to less soft tissue augmentation compared with CTG.	Faster degradation/resorption Lower tissue volume maintenance Variable integration	Minimal resorption Superior volume maintenance Stable soft tissue thickness	Reduced soft tissue thickness over time Less ideal for thick tissue augmentation Limited benefit in large defects	High	[409,450]
Lower biological integration	Some polymers show limited cell infiltration and vascularization compared with autogenous tissue.	Slower and less complete integration Limited vascularization in some materials May act as foreign body initially	Native tissue with complete integration Rapid revascularization Natural remodeling	Delayed maturation of soft tissue Possible fibrous encapsulation Less natural tissue blending	Low	[214,215,216,217,218,219,220,221,222,245,246,247,248,249,250,251]
Lower effectiveness in severe defects	Less effective than CTG in deep recessions, thin phenotypes, and large or complex defects.	Limited root coverage in RT2/RT3 Not ideal for advanced recession Less benefit in multiple recessions	Gold standard for severe defects Better root coverage outcomes Superior esthetic results	Not suitable as a universal substitute CTG still required in complex cases Risk of under-treatment	High	[409,418,419,420,421]
Material variability	Properties may vary between brands, batches, and formulations.	Variable composition and processing non-standardized manufacturing Differences in degradation behavior	Consistent biological properties Patient-specific but well predictable No batch-to-batch variability	Difficult outcome prediction Need for material-specific knowledge Different handling characteristics	Low	[451]
Cost	Advanced polymeric biomaterials may be expensive and not always cost-effective.	High material cost in many cases Limited availability in some regions Not always reimbursed	No material cost; Widely available; No additional expense	Increased treatment cost Financial barrier for some patients Cost/benefit must be considered	n/a	[451]
Lack of bioactivity (when not functionalized)	Some polymers are biologically inert and do not actively promote regeneration.	Passive scaffolds without signaling Limited stimulation of cell activity Requires functionalization	Contains natural ECM components Biologically active Supports natural healing cascade	Slower healing in non-bioactive materials May require combination therapies Limited regenerative potential	Low	[156,157,158,159,160,161,279,280,281,282,283,284,285,286]
Possible adverse reactions	Risk of foreign body reaction, inflammation, or hypersensitivity (to some polymers).	Risk of inflammatory reaction Possible foreign body response Allergic reactions (rare but possible)	Autologous tissue; No risk of immune rejection; Minimal inflammation	Patient discomfort Need for biocompatibility assessment Potential early failure	Low	[304,305,306,307,308,309,310,311]
Limited regulatory approval	Many polymeric biomaterials still lack long-term clinical approval for periodontal use.	Limited regulatory approval Some materials still experimental Limited clinical guidelines	Long-established clinical use Widely accepted Supported by guidelines	Restricted clinical use Off-label applications Need for further evidence	n/a	[451]
Learning curve and handling	Some biomaterials require specific handling and surgical techniques.	Technique-sensitive handling Requires special storage Variable ease of use	Well-known technique; Easy to handle; High clinical familiarity	Longer learning curve Need for training and experience Risk of application errors	Low	[406,407,447,448,449]

**Evidence support.** Grades the strength of the clinical evidence supporting the stated comparison, using the definitions given beneath Table 7: High, at least one systematic review or meta-analysis of randomized controlled trials in a periodontal or peri-implant soft tissue augmentation indication; Moderate, at least one randomized controlled trial in such an indication, or a systematic review of non-randomized or adjunctive studies; Low, case series, preclinical or in vitro evidence only. Entries marked n/a denote practical, economic or regulatory considerations rather than clinical outcomes, for which the concept of a level of clinical evidence does not apply. These categories are descriptive evidence tiers based on study design and availability; they do not represent a formal certainty-of-evidence assessment (for example, GRADE) or a formal risk-of-bias appraisal. CTG—connective tissue graft; ECM—extracellular matrix; RT—recession type.

**Table 10:** Quantitative comparison of polymer-based substitutes and autogenous grafts across the main soft tissue indications. Values are as reported in the cited sources; direction of difference indicates which material performed better for that outcome. Differences that were not statistically significant are marked accordingly. Values for keratinized tissue width gain and for patient-reported outcomes reflect the consistent direction reported across the cited trials rather than a single pooled estimate.

Indication	Outcome Measure	Polymer-Based Substitute	Autogenous Graft	Direction of Difference	Ref.
Multiple gingival recessions	Mean and complete root coverage	Xenogeneic collagen matrix	Connective tissue graft	Significantly favours autogenous graft	[409]
Multiple gingival recessions	Keratinized tissue width, clinical attachment level	Xenogeneic collagen matrix	Connective tissue graft	No significant difference	[409]
Peri-implant mucosal thickness	Thickness gain at 12 months	0.66 ± 0.58 mm (volume-stable collagen matrix)	1.0 ± 0.75 mm (connective tissue graft)	Favours autogenous graft; non-inferiority design	[450]
Keratinized tissue augmentation	Width gain	Collagen matrix	Free gingival graft	Favours autogenous graft; greater shrinkage with matrix	[418,419,420,421]
All indications	Operating time, postoperative pain, donor-site morbidity	Polymer-based substitute	Autogenous graft	Favours polymer-based substitute	[418,419,450]

**Table 11:** Emerging and future technologies in polymer-based biomaterials for soft tissue regeneration. For each technology or approach, the underlying principle, key features, potential clinical benefits, and current translational challenges are summarized.

Technology/Approach	Description	Key Features	Potential Benefits	Current Challenges	Development Stage	Ref.
Smart polymers (stimuli-responsive materials)	Polymers that respond to internal or external stimuli (pH, enzymes, ROS, temperature, light, magnetic fields) and adapt their behavior.	On-demand drug/growth factor release Adaptive degradation Environmental sensing Self-healing properties	Dynamic control of healing Reduced infection and inflammation Improved tissue integration Personalized and precise therapy	Complex design and manufacturing Safety and long-term stability Limited clinical validation High development cost	Preclinical	[393,394,395,396,397,398,399,400,401,485]
Injectable scaffolds and hydrogels	Liquid or low-viscosity systems that gel in situ and conform to complex defects.	Minimally invasive delivery Conformable to irregular defects Can carry cells and bioactive molecules In situ gelation (thermo-, pH-, light- or enzyme-triggered)	Minimally invasive procedures Complete defect filling Enhanced patient comfort Suitable for peri-implant soft tissues	Mechanical weakness before gelation Burst release of bioactive agents Limited long-term mechanical stability Standardization needed	Early clinical	[486]
3D bioprinting and additive manufacturing	Layer-by-layer fabrication of scaffolds or tissue constructs with controlled architecture.	Precise control of pore size and architecture Patient-specific scaffolds multi-material and multi-layer constructs Incorporation of cells and biomolecules	Customized defect-adapted scaffolds Optimized mechanical and biological performance Enhanced vascularization and integration Reproducible and scalable production	Technical complexity and cost Limited printing resolution for soft tissues Cell viability during printing Regulatory hurdles	Preclinical	[369,370,371,372,373,374,375]
Biofunctionalization and bioactive modification	Chemical modification or incorporation of bioactive moieties to enhance biological performance.	Peptide/growth factor immobilization ECM-mimetic coatings Antimicrobial and anti-inflammatory modification Bioactive nanoparticle incorporation	Enhanced cell adhesion and proliferation Controlled immune response Improved soft tissue integration Reduced risk of infection	Stability of bioactive molecules Controlled release kinetics Potential cytotoxicity Complex regulatory pathways	Preclinical	[169,170,171,172,173,174,175,176,177,178,179,180,181,182,183,184,185,186,187,188,189,190,191,192,193,194,195,196,279,280,281,282,283,284,285,286]
Cell and extracellular vesicle (EV) integration	Use of stem cells, progenitor cells, or EVs to enhance regenerative potential of polymeric scaffolds.	Autologous or allogenic cells/EVs Paracrine signaling and immunomodulation Improved vascularization Enhanced matrix remodeling	Accelerated soft tissue regeneration Improved tissue quality Reduced inflammation Potential for scarless healing	Cell sourcing and expansion Storage and handling of EVs Safety and immunogenicity High cost	Preclinical	[328,329,330,331,332,333,334,335,336,337,338,339,340,341,342,343,344,345,346,347,348]
AI- and machine learning-assisted design	Computational design and optimization of biomaterials using artificial intelligence and big data.	Prediction of material properties Optimization of composition and structure Data-driven design of scaffolds Integration with digital patient data	Faster material discovery Optimized performance Personalized biomaterial design Reduced experimental cost and time	Data availability and quality Algorithm interpretability Need for interdisciplinary expertise Validation in clinical settings	In silico/concept	[487,488]
Smart delivery systems and biosensors	Integration of sensors or controlled-release systems for real-time monitoring and therapeutic delivery.	Real-time monitoring (pH, enzymes, inflammation markers) Closed-loop drug delivery Wireless and implantable sensors Controlled release	Personalized, real-time therapy Early detection of complications Improved clinical outcomes Reduced need for follow-up visits	Miniaturization and biocompatibility Power supply and data transmission Long-term reliability Regulatory approval	In vitro/concept	[376,377,378,379,380,381,382,383,384,385,386,387,388,389,390,391,392,393,394,395,396,397,398,399,400,401]
Personalized and precision biomaterials	Development of patient-specific biomaterials based on genetic, phenotypic, and clinical data.	Integration of patient data (imaging, genomics, phenotype) Customized scaffold composition Personalized degradation profiles Precision medicine approach	Tailored regenerative therapy Higher predictability Better esthetic and functional outcomes Optimized long-term stability	Data integration and privacy High cost and complexity Need for regulatory frameworks Clinical validation required	Preclinical	[489,490,491,492,493]

**Development stage.** Indicates the stage of development reached for each approach in periodontal or peri-implant soft tissue applications: concept or in silico, where the approach has been proposed or modelled but not yet built; in vitro; preclinical, where it has been tested in animal models; and early clinical, where first clinical reports exist. None of the approaches listed has yet been evaluated in randomized controlled trials in these indications. AI—artificial intelligence; ECM—extracellular matrix; EV—extracellular vesicles; ROS—reactive oxygen species.

**Table 12:** Clinical decision tree for selecting polymer-based biomaterials in soft tissue regeneration: A stepwise framework proceeding from defect characterization through treatment objectives, biologic requirements, delivery preference, material properties, and patient factors to the final decision and follow-up. For each step, the relevant clinical consideration, key questions, suggested biomaterial options, and supporting rationale are provided. The algorithm synthesizes the material properties, biological requirements and clinical evidence set out in Section 2, Section 3, Section 4, Section 5, Section 6 and Section 7 and does not introduce new sources; supporting references for each material and indication are given in Table 1, Table 2, Table 3, Table 4, Table 5, Table 6, Table 7, Table 8, Table 9, Table 10 and Table 11 and in the corresponding sections.

Step	Clinical Consideration	Key Questions	Suggested Biomaterial Options	Rationale/Notes
1. Defect characterization	Assess the defect and patient factors.	What is the type and size of the defect? Is keratinized tissue inadequate? Is the phenotype thin or thick? Is this a recession, ridge defect, or peri-implant soft tissue deficiency? What are the patient’s systemic conditions and risk factors?	Small/multiple recessions, thin phenotype: HA, collagen matrix, injectable hydrogels Keratinized tissue augmentation: collagen matrix, acellular dermal matrix Peri-implant soft tissue deficiencies: collagen matrix, HA-based fillers, injectable systems Complex/large defects: autogenous grafting remains the reference approach; composite or cell-based scaffolds remain investigational	Match material to defect type, soft tissue quality, and esthetic demands. Consider patient comorbidities (e.g., diabetes, smoking) that may affect healing.
2. Treatment objectives	Define the primary goal of treatment.	Is the goal root coverage, KTW increase, soft tissue volume, or contour enhancement? Is immediate esthetic improvement required? Is long-term stability critical?	Root coverage + esthetics: collagen matrix + EMD/HA KTW increase: collagen matrix, acellular dermal matrix Volume augmentation: autogenous CTG remains the reference standard for predictable volume stability; selected collagen-based substitutes are an alternative where morbidity is a priority Long-term stability priority: favor approaches supported by long-term clinical evidence; autogenous CTG remains the reference standard, with crosslinked collagen matrices as an alternative	Different materials offer different balance between esthetics, volume, and biologic integration.
3. Biologic requirements	Determine the desired biologic response.	Is angiogenesis promotion important? Is cell recruitment or immunomodulation desired? Is controlled degradation necessary?	Pro-angiogenic/cell recruitment: HA, chitosan, fibrin-based systems Immunomodulatory/anti-inflammatory: chitosan, collagen, HA Controlled degradation: crosslinked collagen, synthetic blends (e.g., PLGA, PCL)	Select materials with documented biological properties that support vascularization, fibroblast activity, and immune balance.
4. Delivery preference	Choose the most appropriate delivery form.	Is a minimally invasive approach preferred? Is the site accessible for flap surgery? Is handling ease or injectability important? Is a scaffold or injectable system more suitable?	Injectable systems (HA gels, nanoparticles, thermosensitive hydrogels): minimally invasive Membranes/matrices: when space maintenance or coverage is required Prefabricated scaffolds: for larger volume needs or complex topography	Delivery mode affects patient comfort, surgical complexity, and adaptation to defect morphology.
5. Material properties	Evaluate material properties and evidence.	What is the resorption rate? What are the mechanical properties? Is the material biofunctionalized? What is the level of clinical evidence?	Fast-resorbing: HA, gelatin, fibrin Slow-resorbing/stable: crosslinked collagen, PLGA, PCL Biofunctionalized: peptide-modified, growth factor-loaded, or ECM-derived materials Highest clinical evidence: collagen matrices; moderate clinical evidence: HA	Balance degradation, mechanical support, and bioactivity with clinical evidence and predictability of outcomes.
6. Patient factors and preferences	Consider patient-specific factors.	Is the patient concerned about donor site morbidity? Are there financial considerations? What are patient expectations?	Patients refusing donor site: polymer-based alternatives High esthetic demand: prioritize materials with the strongest indication-specific clinical evidence; biofunctionalized materials remain investigational Cost-sensitive cases: consider local material and procedural costs; formal cost-effectiveness evidence remains limited	Patient-centered decision-making improves satisfaction and treatment adherence.
7. Final decision and follow-up	Implement and monitor.	Is proper handling and placement ensured? Is the material protected during healing? Is follow-up monitoring scheduled?	Follow manufacturer’s protocol for handling and application Protect the surgical site from mechanical trauma according to the procedure and manufacturer’s instructions Monitor healing and tissue maturation regularly	Proper application and follow-up are essential for achieving predictable outcomes.

**Status of the options listed.** Experimental or preclinical options are explicitly identified as such and should not be interpreted as recommendations for routine clinical use. Where clinical evidence is limited, the framework indicates the direction of current thinking rather than an established standard of care, and autogenous grafting remains the reference approach for the indications in which it is identified as such. The algorithm has not been prospectively validated (Section 7.5). ECM—extracellular matrix; EMD—enamel matrix derivative; HA—hyaluronic acid; KTW—keratinized tissue width; PCL—polycaprolactone; PLGA—poly(lactic-co-glycolic acid).

## Data Availability

Not applicable. No new data were created or analyzed in this study; data sharing is not applicable to this article.

## Abbreviations

The following abbreviations are used in this manuscript:

CTG	Connective Tissue Graft
FGG	Free Gingival Graft
ECM	Extracellular Matrix
GTR	Guided Tissue Regeneration
GBR	Guided Bone Regeneration
PDL	Periodontal Ligament
PDLSC	Periodontal Ligament Stem Cell
MSC	Mesenchymal Stem Cell
GMSC	Gingiva-derived Mesenchymal Stem Cell
HA	Hyaluronic Acid
PRF	Platelet-Rich Fibrin
A-PRF	Advanced Platelet-Rich Fibrin
i-PRF	Injectable Platelet-Rich Fibrin
CGF	Concentrated Growth Factor
PDGF	Platelet-Derived Growth Factor
TGF-β	Transforming Growth Factor-beta
VEGF	Vascular Endothelial Growth Factor
FGF-2	Fibroblast Growth Factor-2
BMP	Bone Morphogenetic Protein
rhBMP-2	Recombinant Human Bone Morphogenetic Protein-2
EGF	Epidermal Growth Factor
IGF	Insulin-like Growth Factor
PCL	Polycaprolactone
PLA	Polylactic Acid
PGA	Polyglycolic Acid
PLGA	Poly(lactic-co-glycolic acid)
PEG	Polyethylene Glycol
PVA	Polyvinyl Alcohol
GelMA	Gelatin Methacryloyl
EV	Extracellular Vesicle
SHED	Stem cells from Human Exfoliated Deciduous teeth
ADSC	Adipose-Derived Stem Cell
RGD	Arginine–Glycine–Aspartate (peptide motif)
AI	Artificial Intelligence
ML	Machine Learning
CT	Computed Tomography
CBCT	Cone-Beam Computed Tomography
